# AMPA receptor trafficking and its role in heterosynaptic plasticity

**DOI:** 10.1038/s41598-018-28581-w

**Published:** 2018-07-09

**Authors:** G. Antunes, F. M. Simoes-de-Souza

**Affiliations:** Center for Mathematics, Computation and Cognition, Federal University of ABC, São Bernardo do Campo, SP Brazil

## Abstract

Historically, long-term potentiation (LTP) and long-term depression (LTD), the best-characterized forms of long-term synaptic plasticity, are viewed as experience-dependent and input-specific processes. However, cumulative experimental and theoretical data have demonstrated that LTP and LTD can promote compensatory alterations in non-stimulated synapses. In this work, we have developed a computational model of a tridimensional spiny dendritic segment to investigate the role of AMPA receptor (AMPAR) trafficking during synaptic plasticity at specific synapses and its consequences for the populations of AMPAR at nearby synapses. Our results demonstrated that the mechanisms of AMPAR trafficking involved with LTP and LTD can promote heterosynaptic plasticity at non-stimulated synapses. These alterations are compensatory and arise from molecular competition. Moreover, the heterosynaptic changes observed in our model can modulate further activity-driven inductions of synaptic plasticity.

## Introduction

Long-term forms of synaptic plasticity are persistent modifications in the efficacy of the synaptic transmission^1^. Long-term depression (LTD) and long-term potentiation (LTP) are the best-characterized forms of synaptic plasticity and strong candidates to underlie learning and memory^1^. LTP is a long-lasting increase in the synaptic strength^1^. LTD consists of a persistent reduction of the synaptic weight^1^. Historically, LTP and LTD are regarded as experience-dependent and input-specific events^2^. The input-specificity of LTP and LTD is consistent with Hebbian-type learning rules and the cellular mechanisms for associative memory^3^.

At glutamatergic synapses, LTP and LTD require the activation of several signalling pathways enclosed in the dendritic spines^2^. Mature dendritic spines are formed by a bulbous head connected to the parental dendrite through a narrow spine neck that imposes a diffusion barrier to the flow of molecules and ions in and out of the spine head^4^. The structure of the dendritic spines confines the molecules involved with the excitatory postsynaptic transmission and synaptic plasticity^2,4^ and contributes to the notion that LTP and LTD are input-specific^5^.

For several years, theoretical works have indicated that Hebbian-rules of learning alone are unstable^3,6^. The increase of the synaptic strength consequent to LTP increases the probability that the same synapse will be further potentiated in a continuous loop^3^. LTD increases the likelihood that the same synapse will be further depressed until eventually reaching a synaptic weight of zero^3^. Thus, theoretical works have postulated the existence of compensatory mechanisms that stabilize the occurrences of LTP and LTD^3^. However, the biological processes that underlie such compensatory alterations remain poorly understood. Slow forms of synaptic scaling that affect the whole neuron and take several hours to occur have been extensively investigated^6,7^. Several works have also indicated the existence of faster and more localized events that balance the synaptic weights among few synapses^3^. For instance, the potentiation of multiple spines on a single dendrite segment causes the shrinkage and synaptic weakening of neighbouring non-stimulated dendritic spines^8^. The induction of spike-timing dependent plasticity at one synapse affects nearby synapses in a compensatory manner^9^. In addition, the potentiation of a single synapse lowers the threshold for the induction of LTP at vicinal synapses^10^. Experimental evidence suggests that such heterosynaptic alterations might involve competition for limited resources, activation of specific signalling mechanisms and the diffusion of active enzymes^8,11–13^.

Among the molecules that underlie synaptic plasticity, AMPA-type ionotropic glutamatergic receptors (AMPARs) play a central role in both LTP and LTD^14^. In neurons, AMPARs are highly mobile and undergo constitutive and activity-dependent trafficking^15–17^. Changes in the number of synaptic AMPARs are crucial events during experience-dependent synaptic modifications^14,16^. At the synapses between pyramidal CA3 and CA1 hippocampal neurons, LTP involves an increase in the number of synaptic AMPARs in an activity-dependent manner^14^. In contrast, LTD requires the reduction of synaptic AMPAR clusters^14^. However, it is unclear whether changes in the number of AMPARs at one synapse affect adjacent synapses in compensatory manners. In this work, we developed a computational model of a tridimensional spiny dendritic segment to study the dynamics of the constitutive and activity-driven AMPAR trafficking. We used the model to investigate whether activity-dependent changes in the population of AMPARs of specific dendritic spines regulate the synaptic strength of non-stimulated vicinal synapses. Our results revealed that the mechanisms of AMPAR trafficking can promote heterosynaptic plasticity and, consequently, contribute to the stabilization of the neuronal activity during occurrences of synaptic plasticity.

## Results

### Modelling the constitutive trafficking of AMPARs

**Figure 1:**
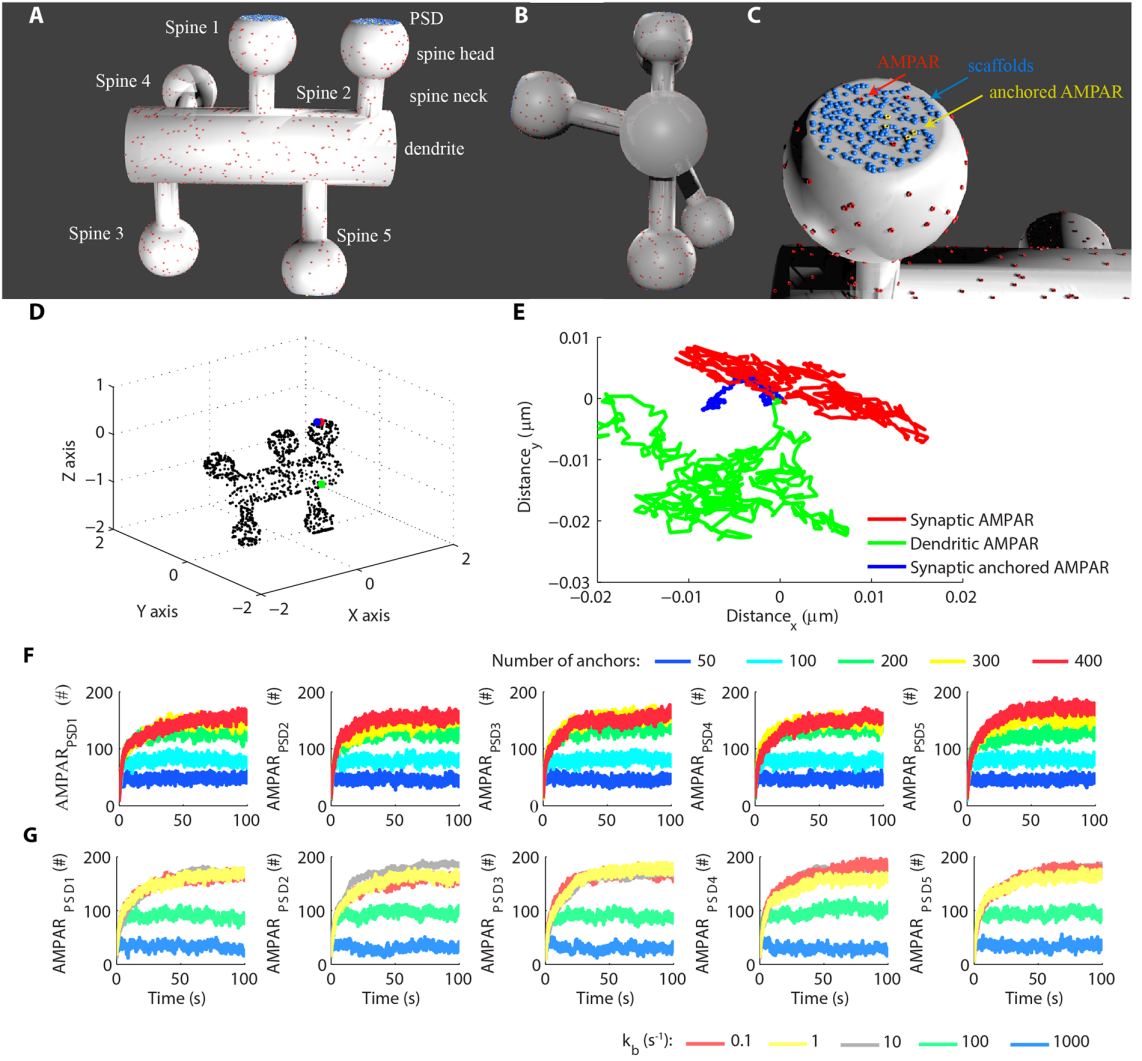
Tridimensional computational model of AMPAR trafficking. **(A)** Overall view of the tridimensional dendritic segment containing five dendritic spines. Each spine consisted of a neck, a head, and a PSD on the top of the head. **(B)** Lateral view of the dendritic segment. **(C)** At each PSD, we simulated scaffold proteins that interacted with AMPARs and anchored them. **(D)** Distribution of AMPARs along the mesh. **(E)** Trajectories of three distinct AMPARs during 5 ms of simulated time. There is a receptor on the dendrite, a free synaptic receptor and a synaptic receptor associated to a scaffold. Their initial positions are indicated in **(D)**, and their initial coordinates were set as (0,0) for visualization of the trajectories. **(F)** The initial number of available scaffold molecules (anchors) regulated the number of synaptic AMPARs at each PSD. **(G)** The rate constant for the dissociation of AMPARs from the scaffold molecules controlled the number of synaptic receptors stabilized at each PSD (300 scaffolds per synapse).

To investigate the dynamic regulation of AMPARs, we developed a tridimensional mesh in CellBlender/MCell^18–20^ to simulate a small dendritic segment containing 5 dendritic spines (Fig. 1A,B). We assigned the top of each spine head as its postsynaptic density (PSD), which is a protein-rich layer that concentrates the molecular machinery involved with postsynaptic glutamatergic transmission^2^.

AMPARs diffuse randomly on extra-synaptic membranes^15^. However, they accumulate on the synaptic membranes due to interactions with scaffold molecules^16^. For simplicity, we assumed that each receptor interacts with a single scaffold molecule simulated with a second-order reaction of complex formation and a first-order reaction of complex dissociation. We simulated a population of scaffold molecules at each PSD to trap AMPARs^16,17^ by forming *scaffold*.*AMPAR* complexes (Fig. 1C, indicated as anchored AMPAR). Free AMPARs were simulated as highly mobile particles^15^. However, synaptic receptors had more restricted trajectories due to their interactions with scaffold molecules, which were confined at the synapses during the simulations. Figure 1D and E show examples of the trajectories of AMPARs in different locations of the model.

We first verified the gradual accumulation of AMPARs at the synapses starting from random distributions of 1000 receptors throughout the model surface (Suppl. Fig. S1), excluding the two lateral extremities of the dendritic segment, which represented lateral cross-sections. The basal number of synaptic AMPARs is highly variable and affected by the occurrences of synaptic plasticity^14^. However, it has been estimated that an average synapse contains around 100 receptors^21^. Thus, our initial goal was to obtain a population of approximately 100 receptors per synapse. Hippocampal pyramidal neurons have large amounts of scaffold molecules^22^ that regulate the number of anchored AMPARs^14,16,17^. We performed simulations varying the number of scaffold molecules per spine and their affinity for AMPARs to verify the impact of these two parameters in the populations of synaptic AMPARs of the model^14,17^. Our results demonstrated that populations of 50, 100 and 200 scaffolds per PSD greatly affected the total number of synaptic AMPARs (Fig. 1F). However, larger populations of scaffolds (300 and 400 copies) had less pronounced effects, which indicated that the number of AMPARs available for binding was the limiting factor for these amounts of scaffold molecules. Next, we investigated how the affinity for the interaction between AMPARs and the scaffold proteins regulated the population of synaptic receptors. We performed simulations with 200 (Suppl. Fig. S2) and 300 (Fig. 1G) scaffolds per PSD and systematically changed the first-order rate (k_b1_) for the reaction of dissociation of the complex *scaffold*.*AMPAR*. The second-order rate constant of complex association was kept constant during all simulations (k_f1_ = 1 µm^2^.molecule^−1^.s^−1^). A k_b1_ of 100 s^−1^ resulted in approximately 100 receptors per synapse containing 300 scaffold molecules that we set as our initial control condition (Fig. 1G).

**Figure 2:**
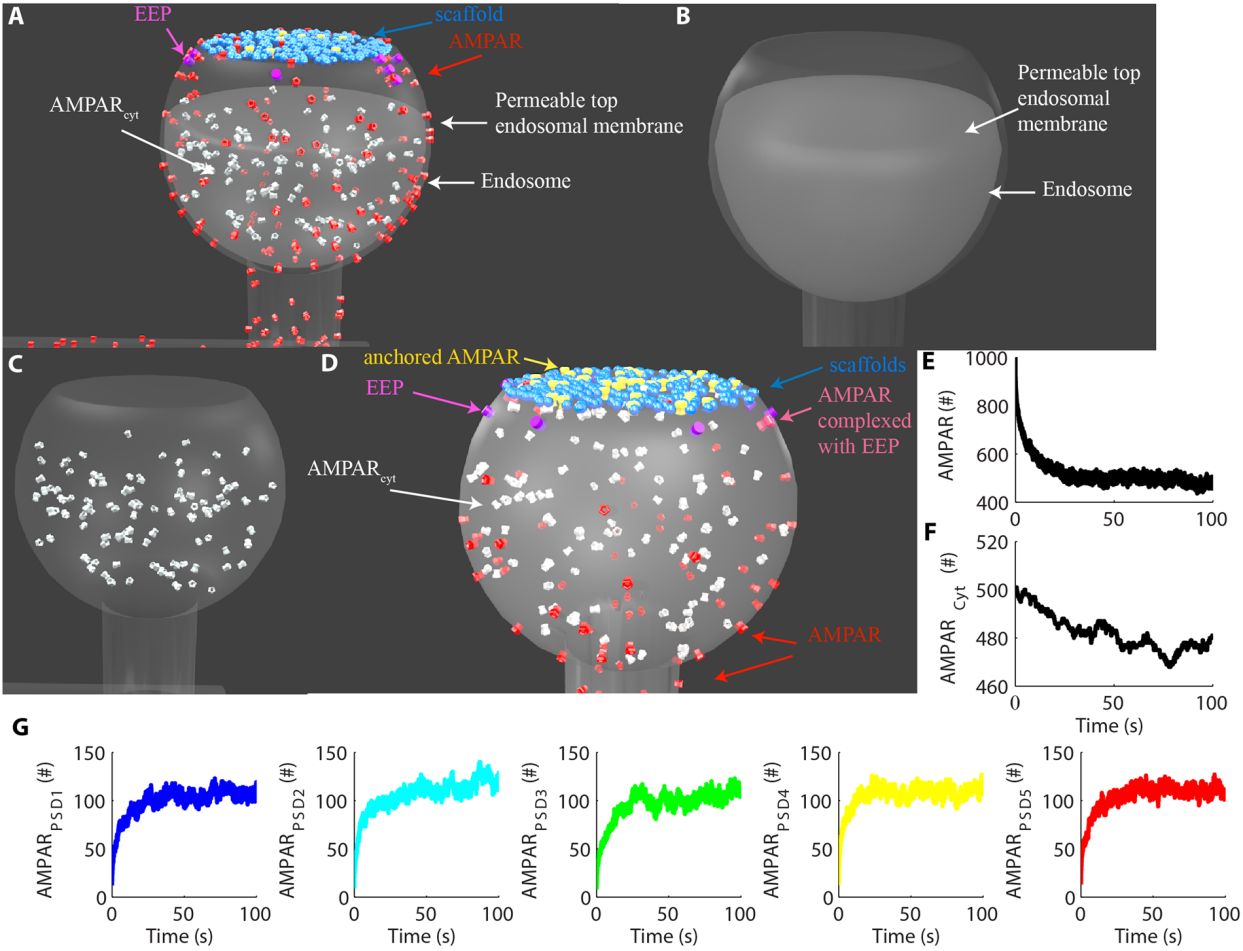
AMPAR trafficking model. **(A)** To simulate the endocytosis and exocytosis of AMPARs, we implemented a single EZ per spine where we placed 10 EEPs to catalyze the internalization and externalization of AMPARs. **(B)** We simulated a single large endosome inside each spine head. The top membrane of each endosome was permeable to cytoplasmic AMPARs (AMPAR_cyt_) to allow their interaction with EEP. The impermeable bottom of the endosomes confined the cytoplasmic molecules inside the spine heads. **(C)** Detailed view of AMPAR_cyt_ in the cytoplasm of the spine head. **(D)** Molecular components of the model and their distributions. In (**C,D**), the endosomes were rendered transparent for better visualization of the intracellular molecules. **(E)** Time course of the total amount of free AMPARs. We released 1000 free AMPARs at the beginning of the simulations to verify their accumulations at the synapses. **(F)** Total number of AMPAR_cyt_ obtained by the balance of exocytosis and endocytosis of receptors. **(G)** Number of AMPARs at each PSD combining free and anchored receptors.

Next, we implemented the constitutive endocytosis and exocytosis of AMPARs using a pool of cytosolic AMPARs per dendritic spine released from a single large endosome (Fig. 2A,B). We simulated the exocytosis and endocytosis of AMPARs using a single endocytic/exocytic enzyme (EEP), which we distributed randomly at an endocytic zone (EZ) that surrounded the PSD of each spine (see methods for further details) (Fig. 2C,D). We then examined the distribution of AMPARs in synaptic, extra-synaptic and cytosolic compartments starting from random distributions of 1000 receptors on the model membrane, and 500 intracellular receptors (100 cytosolic AMPARs per spine) (Fig. 2E,F). After reaching steady-state, each synapse contained a population of approximately 100–120 AMPARs including both free and anchored receptors (Fig. 2G).

### Activity-dependent changes in the populations of synaptic AMPARs

**Figure 3:**
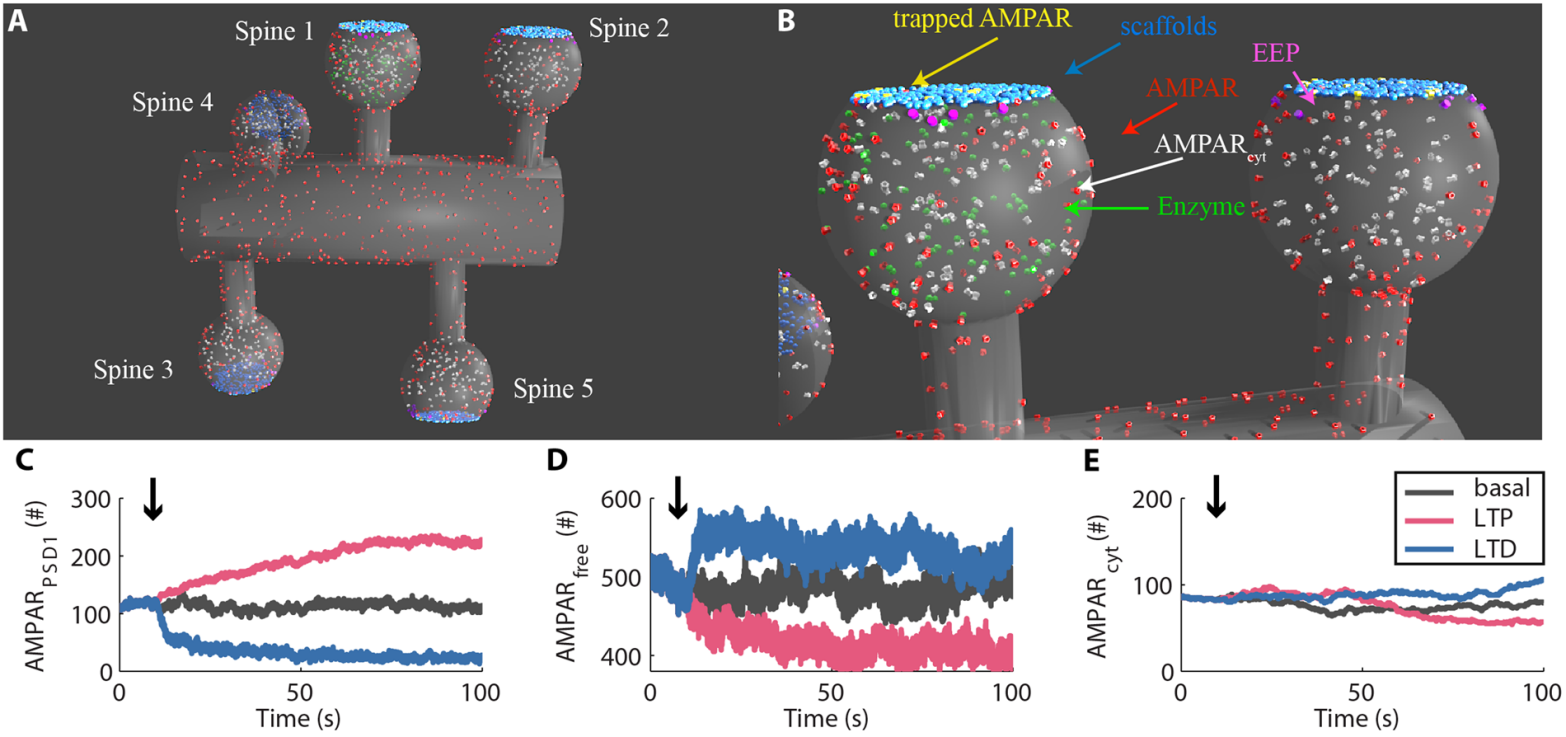
Activity-dependent changes of synaptic AMPARs. We implemented two enzymes that phosphorylate the scaffold molecules to simulate LTP and LTD. **(A)** View of the whole mesh used in the simulations with the enzymes involved with synaptic plasticity released into the spine 1 (in green). **(B)** Detailed view of the enzymes released into the spine 1 to induce synaptic plasticity through the phosphorylation of scaffold molecules. In (**A,B**), we rendered the endosomes transparent for better visualization of the intracellular molecules. **(C–E)** Time courses of synaptic AMPARs at rest and during simulations of LTP and LTD at PSD1 (spine 1) **(C)**, the total population of free AMPARs (AMPAR_free_) on the dendritic segment simulated **(D)** and the population of intracellular AMPARs inside the spine 1 **(E)**.

Next, we investigated the dynamics of activity-driven AMPAR trafficking. The occurrence of hippocampal LTP is associated with an increase in the number of synaptic AMPARs^14^. In contrast, hippocampal LTD involves a reduction of the population of synaptic AMPARs^14^. The exact mechanisms that promote the changes in the number of synaptic AMPARs during LTP and LTD are not completely established. Nevertheless, several pieces of evidence have indicated that LTP and LTD involve changes in the affinities of scaffold molecules for interacting with AMPARs consequent to the phosphorylation of specific residues catalyzed by protein kinases^14,16,17^. For simplicity, we implemented two generic cytosolic enzymes (Fig. 3A,B), one associated with LTP (enzLTP), and the other with LTD (enzLTD). Thus, our model of synaptic plasticity is a three-state system (basal, LTP, and LTD). Several models simulate synaptic plasticity using two-states systems (LTP and LTD) in which phosphorylation represents LTP and dephosphorylation represents LTD^23,24^. However, recent data demonstrated that LTP and LTD involve activity-driven phosphorylations of different targets^25^. LTP and LTD require post-translational modifications of scaffold molecules and several residues of AMPAR^14,26,27^. Nevertheless, we opted to simulate only the phosphorylation of the scaffold molecules^26,27^ to ensure spatial specificity as they do not diffuse from one synapse to another in our model. The phosphorylation of scaffolds by enzLTP enhanced their affinity to interact with AMPARs by 10-fold^28^. In contrast, the phosphorylation of scaffold molecules catalyzed by enzLTD caused a 10-fold reduction in their affinity for AMPARs^28^.

We released 100 copies of enzLTP in the cytosol of a single spine to simulate LTP and verified how it changes the number of synaptic AMPARs, the number of receptors of the dendritic pool and the number of intracellular AMPARs. Note that the time courses of our simulations correspond only to the initial minutes of synaptic plasticity occurrences. Our results showed that the simulation of enzLTP inside a single dendritic spine increased its population of synaptic AMPARs (Fig. 3C). Moreover, most of the newly inserted receptors came from the dendritic pool (Fig. 3D). Only a few receptors came from the cytosolic pool (Fig. 3E). This result is consistent with experimental data that have shown that the majority (70–90%) of the new inserted AMPARs that reach the PSD through lateral diffusion come from the dendritic pool^29,30^.

Next, we simulated LTD by releasing 100 copies of enzLTD inside a single dendritic spine. Our results indicated that the induction of LTD promoted the dispersion of the synaptic receptors preferentially by lateral diffusion instead of internalization (Fig. 3C,E). Experimental results have demonstrated that LTD requires the endocytosis of AMPARs^14^. Nevertheless, only a small fraction of AMPARs are internalized directly from perisynaptic areas^31^. The majority of receptors appear to be internalized from dendritic and somatic compartments, which suggests that AMPARs are first removed from the synapses through lateral diffusion and internalized in a posterior step. Thus, likely the dendritic pool of receptors acts as an initial fate of receptors removed from a synapse during synaptic plasticity as indicated by our results, while the intracellular pool plays a later role stabilizing the initial change.

### AMPAR trafficking can promote heterosynaptic plasticity

**Figure 4:**
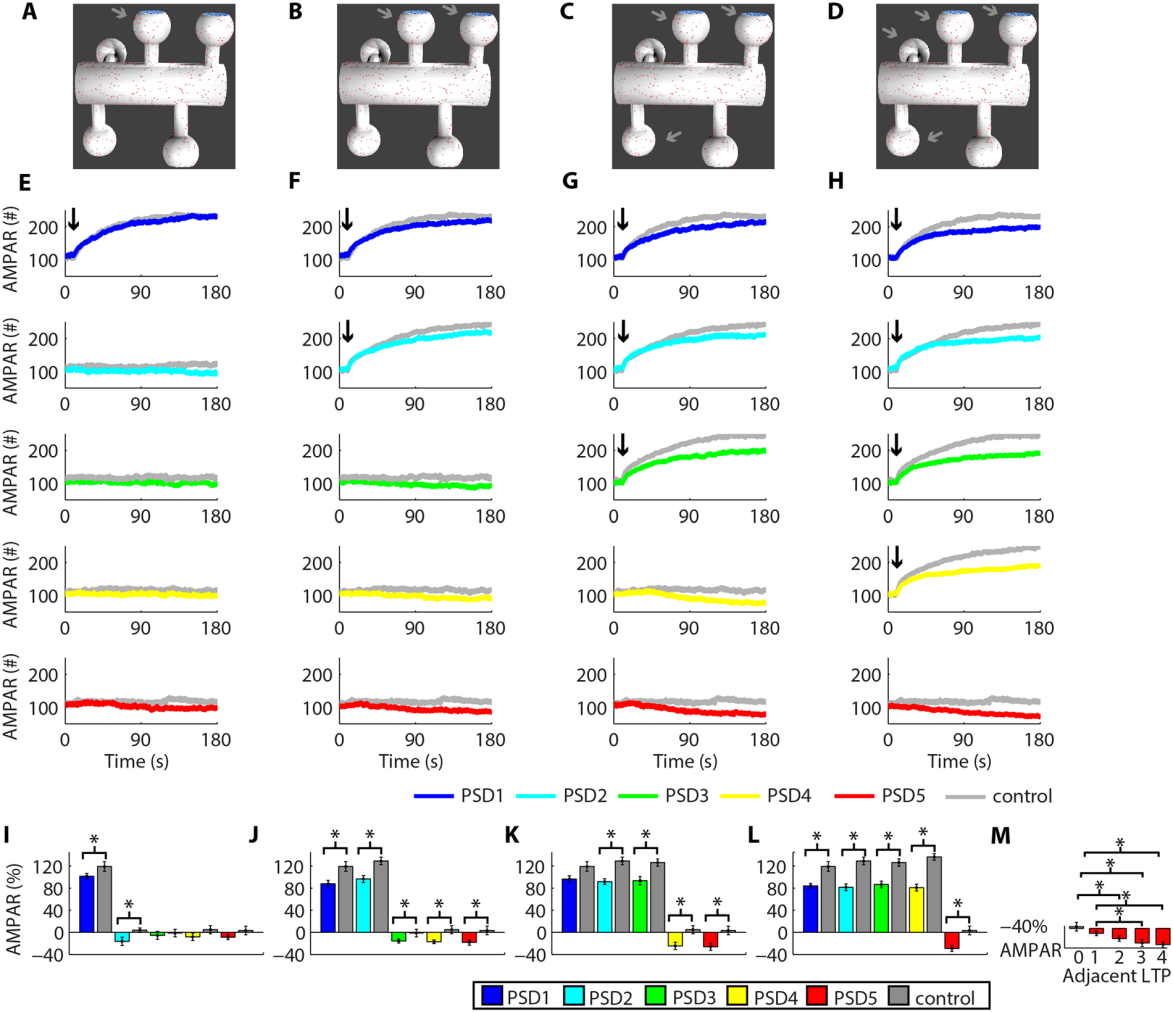
Activity-driven LTP causes heterosynaptic depression. **(A–D)** Synapses with induced LTP. **(E–H)** Time courses of the changes in the number of AMPARs at each PSD simulated during LTP at PSD1 **(E)**, PSD1 and PSD2 **(F)**, PSD1-PSD3 **(G)**, and PSD1-PSD4 **(H)**. The controls correspond to simulations without the induction of synaptic plasticity (Suppl. Fig. S3) and LTP at single synapses (Suppl. Fig. S4). **(I–L)** Changes of AMPARs at each PSD from t_1_ = 10 s to the end of the simulations caused by LTP at one **(I)**, two **(J)**, three **(K)** and four synapses **(L)**. The asterisks indicate statistically significant differences (P < 0.05) for paired T-test comparisons with control simulations. **(M)** The number of vicinal LTP modulated the amplitude of heterosynaptic depression at PSD5. The asterisks indicate statistically significant Tukey post hoc comparisons (P < 0.05).

Since LTP and LTD in our model relied heavily on the lateral diffusion of receptors, the next stage of our work investigated whether the activity-driven trafficking of AMPARs can affect the synaptic weights of adjacent synapses. We simulated LTP at a single synapse (Fig. 4A), simultaneously at two synapses (Fig. 4B), three (Fig. 4C), and four synapses (Fig. 4D) and verified how each of these scenarios regulated the synaptic strength of the nearby non-stimulated synapses (Fig. 4E–H) by measuring the variation of AMPARs at each synapse from the moment of LTP induction to the end of the simulations. Our results showed that the increase in the number of synaptic AMPARs of the dendritic spines undergoing LTP promoted heterosynaptic compensatory reductions in the number of AMPARs at adjacent non-stimulated spines (Fig. 4E,H). We verified the occurrence of heterosynaptic plasticity by comparing the results obtained to control simulations without the induction of synaptic plasticity (Fig. 4I–L, Suppl. Fig. S3). For instance, LTP at PSD1 caused a reduction of 16.3% ± 7.5, n = 6, in the population of AMPAR at PSD2. In contrast, the population of AMPAR at PSD2 varied 3.8% ± 4.0, n = 5, in control simulations without synaptic plasticity (P < 0.05, T-test). Simultaneous occurrences of LTP at PSD1 and PSD2 promoted a reduction of synaptic AMPARs at PSD3 of 15.8% ± 3.7, n = 7. Control simulations without synaptic plasticity at nearby spines exhibited a variation of AMPARs at PSD3 of −1.38% ± 6.7 (n = 5, P < 0.05, T-test). LTP at PSD1, PSD2, and PSD3 led to a decrease of AMPAR at PSD4 and PSD5 of 24.6% ± 6.55 (n = 6, control variation: 4.63% ± 7.23, n = 5, P < 0.05, T-test) and 26.2% ± 6.3 (n = 6, control variation: 3.19% ± 7.7, n = 5, P < 0.05, T-test), respectively. We also observed that the simultaneous occurrences of activity-driven LTP at multiple synapses reduced the maximum potentiation observed. This result was verified by comparisons with control simulations of LTP at single dendritic spines (Fig. 4I–J, Suppl. Fig. S4). The single occurrence of LTP at PSD1 increased its AMPAR population by 119% ± 6.8, n = 5, but the simultaneous occurrence of LTP at PSD1, PSD2, and PSD3 increased the number of AMPARs at PSD1 by 96.3% ± 6 (n = 6, P < 0.05, T-test). Moreover, by measuring the magnitude of the heterosynaptic depression at PSD5 caused by LTP at 1–4 vicinal synapses (Fig. 4M), we confirmed that the number of synapses undergoing LTP regulated the intensity of heterosynaptic depression. For instance, LTP at PSD1 and PSD2 reduced the number of AMPARs at PSD5 by 18.4% ± 4.8 (n = 7, control: 3.19% ± 7.7, n = 5, P < 0.05, T-test). In contrast, simultaneous LTP at PSD1–4 decreased the number of AMPARs at PSD5 by 29.4% ± 5.35 (n = 8, control: 3.19% ± 7.7, n = 5, P < 0.05, T-test). One-way ANOVA (F(4,27) = 10.61, P < 0.01) followed by a Tukey post hoc test indicated a statistically significant difference between groups of heterosynaptic plasticity at PSD5 caused by adjacent LTPs (Fig. 4M). Note, however, that a portion of the receptors inserted at the synapses undergoing LTP came from the cytosolic pool and from the dendritic shaft. Consequently, an increase of 100% in the weight of a single synapse did not produce a reduction of 25% in the AMPAR population of each of its four neighbouring dendritic spines.

**Figure 5:**
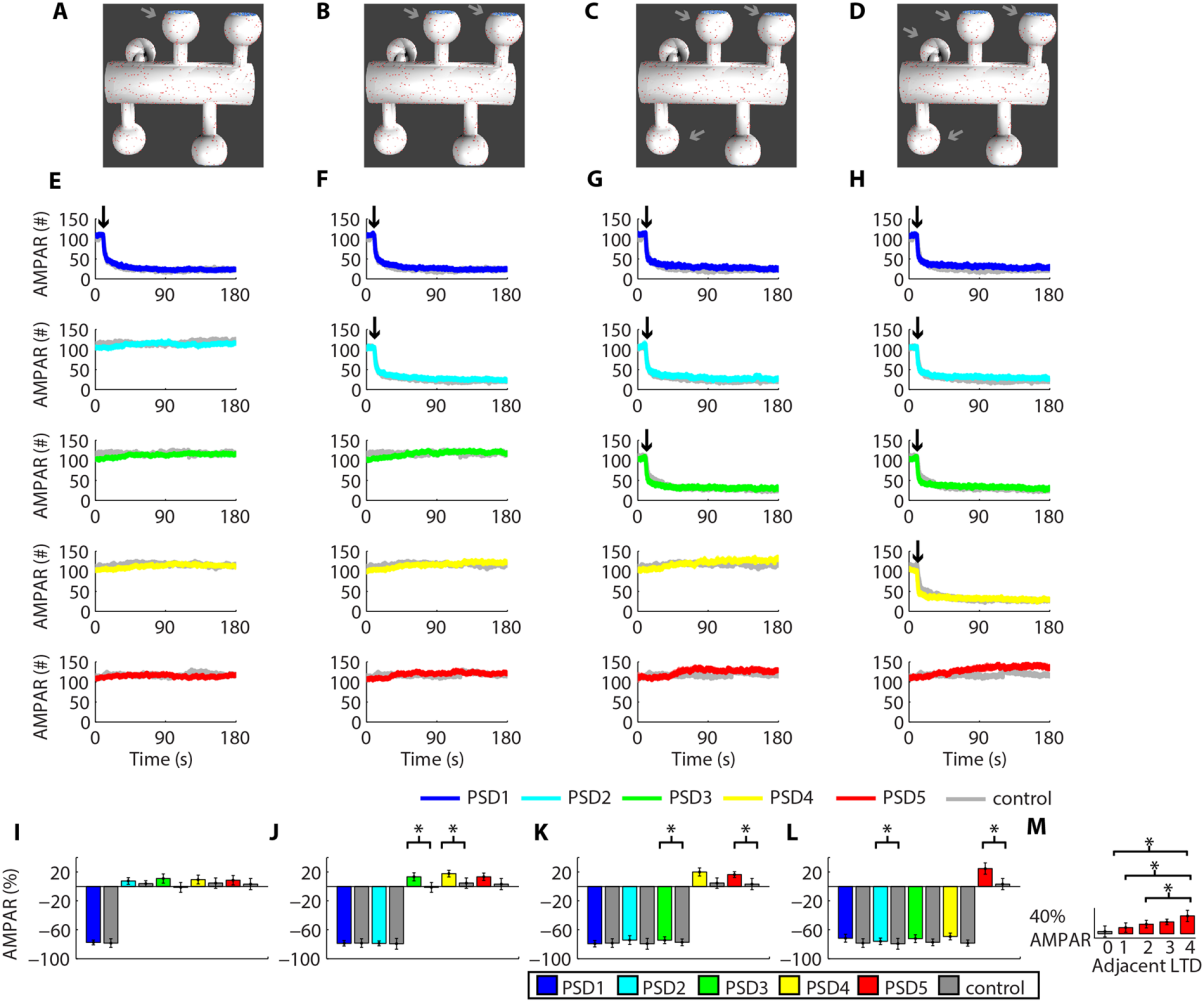
Heterosynaptic potentiation induced by LTD. **(A–D)** Visualization of the dendritic spines with simulated LTD. **(E–H)** Time courses of the changes in the number of AMPARs at each PSD caused by LTD at PSD1 **(E)**, PSD1 and PSD2 **(F)**, PSD1-PSD3 **(G)**, and PSD1-PSD4. The arrows show the moment of LTD induction. The controls are simulations without the induction of synaptic plasticity (Suppl. Fig. S3) or simulations of LTD at single synapses (Suppl. Fig. S5). **(I–L)** Alterations of AMPARs at each PSD estimated from t_1_ = 10 s to the end of the simulations caused by one **(I)**, two **(J)**, three **(K)** and four synapses undergoing LTD simultaneously **(L)**. Asterisks indicate statistically significant T-test comparisons (P < 0.05) with control simulations. **(M)** The number of nearby LTD regulated the magnitude of heterosynaptic potentiation at PSD5. The asterisks indicate statistically significant Tukey post hoc comparisons (P < 0.05).

Next, we verified whether the induction of LTD promoted heterosynaptic plasticity at nearby synapses (Fig. 5A–D). Figure 5E–H shows the time courses for the occurrences of LTD at specific synapses and their effects in vicinal dendritic spines. The changes in the populations of synaptic AMPARs during LTD promoted heterosynaptic plasticity of non-stimulated adjacent synapses (Fig. 5I–L). For instance, LTD at PSD1 and PSD2 increased the population of AMPARs at PSD3 and PSD4 by 13.3% ± 5.5 (n = 7, control: −1.38% ± 6.8, n = 5, P < 0.05, T-test) and 17.7% ± 4.54 (n = 7, control: 4.63% ± 7.23, n = 5, P < 0.05, T-test), respectively. The magnitudes of the heterosynaptic alterations observed were regulated by the number of synapses undergoing LTD simultaneously (Fig. 5M). LTD at PSD1–3 increased the number of AMPARs at PSD5 by 13.4% ± 5.2 (n = 6, control: 3.19% ± 7.7, n = 5, P < 0.05, T-test), but LTD at PSD1–4 increased the population of AMPARs at PSD5 by 24.8% ± 7.8 (n = 7, control: 3.19% ± 7.7, n = 5, P < 0.05, T-test). One-way ANOVA (F(4,30) = 5.23, P = 0.002) followed by a Tukey post hoc test indicated statistically significant differences between groups of heterosynaptic plasticity at PSD5 regulated by different number of adjacent synapses undergoing LTD (Fig. 5M).

Next, we performed simulations using different numbers of scaffold molecules (200 and 100; the control was 300) and free AMPARs released at the beginning of the simulations (1500, 2000, and 2500; the control was 1000) to verify the sensitivity of our model (Suppl. Fig. S6). These changes affected the number of synaptic AMPARs at rest and the magnitudes of LTP and LTD. The reduction of the number of scaffolds from 300 to 100 suppressed heterosynaptic potentiation during LTD at adjacent synapses due to the saturation of the scaffolds. However, the reduction of the number of scaffolds from 300 to 100 molecules per synapse only suppressed heterosynaptic depression caused by LTP at vicinal synapses for versions of the model with high numbers of free AMPARs (1500 to 2500 free receptors released at the beginning of the simulations). We also tested whether simulations with a continuous flow of AMPARs through the lateral membranes could affect the occurrences of heterosynaptic plasticity. For these simulations, we observed substantial variations in the number of synaptic AMPARs among the spines simulated at rest. Nevertheless, simulations of LTP and LTD at specific synapses still caused heterosynaptic changes at nearby dendritic spines verified by visual inspection (Suppl. Fig. S7).

### Heterosynaptic plasticity can modulate posterior inductions of activity-dependent synaptic plasticity

**Figure 6:**
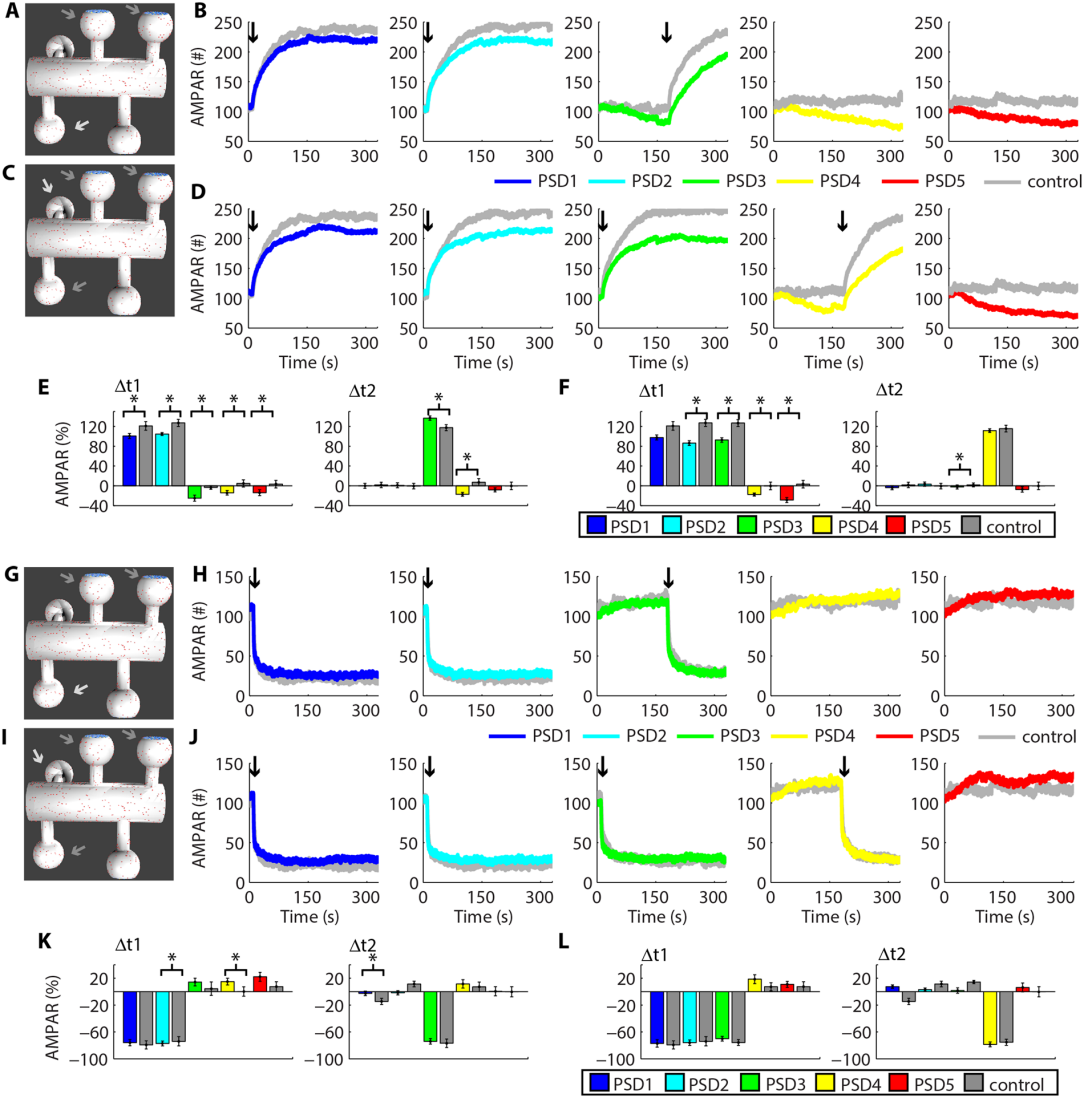
Heterosynaptic plasticity and the further induction of activity-driven synaptic plasticity. Simultaneous occurrences of LTP at two (**A**,**B**) or three synapses **(C,D)** can affect the posterior induction of LTP at a nearby synapse. **(E,F)** Changes of the percentage of AMPARs at each PSD caused by prior (Δt1) and posterior LTP (Δt2). Prior occurrences of LTD at two (**G**,**H**) or three synapses **(I,J)** had little effects on posterior LTD at an adjacent synapse. **(K,L)** Variations of AMPARs at each PSD caused by prior (Δt1) and posterior LTD (Δt2). The arrows in **A**, **C**, **G** and **I** show the synapses stimulated at t1 (10 s, dark arrows) and at t2 (180 s, light arrows). In **B**, **D**, **H**, and **J** the arrows indicate LTP or LTD inductions. All the results of the control simulations are showed in Suppl. Fig. S8. **(E,F**,**K,L)** Asterisks indicate statistically significant T-test comparisons (P < 0.05) with control simulations.

Experimental results revealed that the previous occurrence of LTP facilitates the induction of activity-driven LTP at adjacent dendritic spines^10^. However, the mechanisms that underlie such facilitation are not completely known^10,11^. Thus, we investigated whether the heterosynaptic plasticity observed in our system can regulate further occurrences of activity-driven synaptic plasticity. Initially, we induced LTP at t_1_ (10 s) simultaneously at two (Fig. 6A,B) or three synapses (Fig. 6C,D) to promote heterosynaptic depression at vicinal synapses. Then, we simulated LTP at t_2_ (180 s) at a single nearby synapse (Fig. 6A,C). We measured the change of the populations of AMPAR at each PSD between 10 s and 160 s (Δt1) caused by the first inductions of LTP to verify whether heterosynaptic depression modulated activity-driven synaptic LTP in our system. Next, we measured the variations of AMPARs at each PSD between the induction of posterior LTP (180 s) and the end of the simulations (330 s). This interval corresponded to Δt2 (Fig. 6E,F). The results obtained were compared to control simulations of synaptic plasticity induced at single spines without previous heterosynaptic plasticity. Heterosynaptic plasticity was verified by comparisons to control simulations without synaptic plasticity. Statistical analysis for each induction of LTP demonstrated compensatory changes of the population of AMPARs at non-stimulated synapses in comparison to control simulations without synaptic plasticity at nearby dendritic spines (P < 0.05, T-tests; Fig. 6E,F). Moreover, posterior LTP at PSD3 after prior LTP at PSD1 and PSD2 increased its AMPAR population by 137% ± 4.4 (n = 8, control: 117% ± 6, n = 5, P < 0.05, T-test; Fig. 6E). However, posterior LTP at PSD4 after prior LTP at PSD1–3 increased its number of synaptic AMPARs only by 111% ± 3.8 (n = 12, control: 115% ± 6.84, n = 5, P = 0.22, T-test; Fig. 6F). Nevertheless, the increase of AMPAR at PSD4 was higher than the prior increase of AMPAR at PSD1–3 as determined by ANOVA (F(3,44) = 3.86, P = 0.0155). A Tukey post hoc confirmed a significant difference between the increase of AMPAR at PSD4 and the prior increase of AMPAR at PSD2 (86.2% ± 4.7, n = 12, P < 0.05), and a tendency of difference between PSD4 and PSD3 (127% ± 7.1, n = 5, P = 0.07, Tukey post hoc).

Subsequently, we tested whether the heterosynaptic plasticity caused by the previous LTD of two or three synapses affected the posterior LTD at a nearby synapse (Fig. 6G–J). Our results indicated that previous occurrences of LTD do not modulate posterior LTD in our system. The difference observed between the effect of LTP and LTD in posterior inductions of activity-driven synaptic plasticity results from their different molecular mechanisms in our model. The occurrence of LTD is a first-order process in our system that depends mainly on the change in the affinity between AMPARs and their scaffolds, which is not affected by prior heterosynaptic potentiation. Thus, LTD appears to be less prone to modulations caused by previous heterosynaptic potentiation. In contrast, heterosynaptic depression decreases the number of AMPARs at nearby synapses and increases the number of scaffolds available to interact with receptors in a posterior induction of LTP. The increase in the number of scaffold molecules available, combined with an increase in their affinity for AMPARs during activity-driven LTP, contributed to the overall increase in the magnitude of potentiation observed in our results.

**Figure 7:**
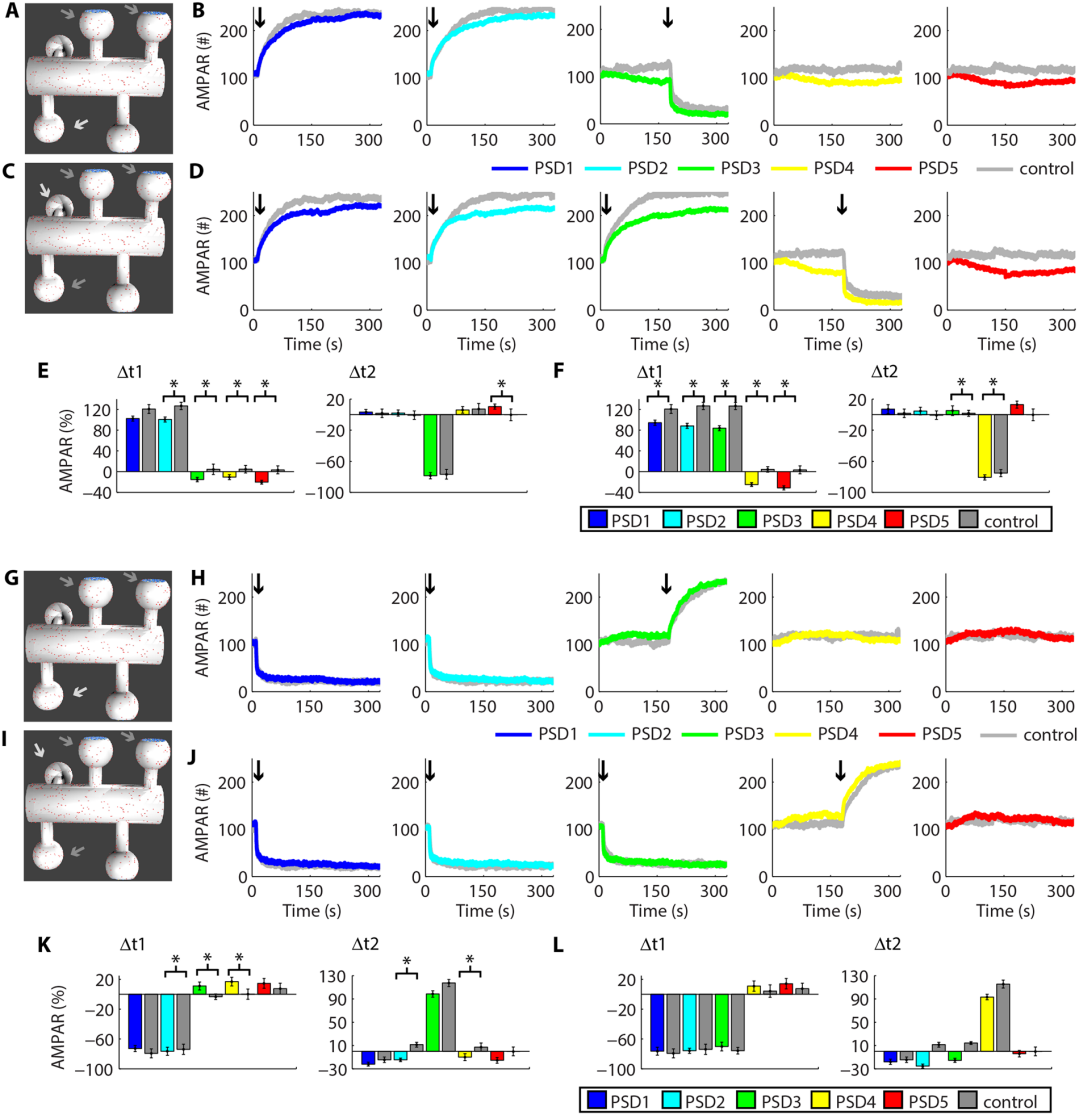
Heterosynaptic plasticity and the posterior induction of activity-driven synaptic plasticity. Prior LTP at two (**A**,**B**) or three synapses **(C,D)** had little effects on the posterior induction of LTD at a vicinal synapse in comparison to control LTD without prior synaptic plasticity **(E,F)**. Alterations of AMPARs at each PSD caused prior LTP (Δt1) and posterior LTD (Δt2). (**G**–**J**) LTD at two (**G**,**H**) or three synapses **(I,J)** did not regulate posterior LTP **(K,L)**. Variations of AMPARs at each PSD for prior LTD (Δt1) and posterior LTP (Δt2). The arrows in **A**, **C**, **G** and **I** indicate the synapses stimulated at t1 (10 s, dark arrows) and at t2 (180 s, light arrows). In **B**, **D**, **H**, and **J** the arrows show the moment of LTP or LTD inductions. **(E,F**, **K,L)** Asterisks indicate statistically significant T-test comparisons (P < 0.05) with control simulations.

Next, we tested whether previous LTP modulated posterior LTD (Fig. 7A–F) and vice versa (Fig. 7G–L). Prior LTP at PSD1–2 promoted a slight tendency of increase of posterior LTD at PSD3 (−78.7% ± 4, n = 10, control: −76.8% ± 6, n = 5, p = 0.44, T-test), but this result was not statistically significant (Fig. 7E,F). However, prior LTP at PSD1–3 significantly increased the magnitude of posterior LTD at PSD4 (−80.8% ± 3.3, n = 11, control: −75.3% ± 4.5, n = 5, P < 0.05, T-test). Though prior LTD appeared to promote a decrease in posterior LTP, our results were not statistically significant (posterior LTP at PSD3: 98.5% ± 5.5, n = 8, control: 117% ± 6, n = 5, P = 0.07; posterior LTP at PSD4: 93.2% ± 4.8, n = 8, control: 115% ± 6.8, n = 5, P = 0.07). All inductions of LTP and LTD promoted heterosynaptic plasticity assessed through comparisons to control simulations (Fig. 7E,F and K,L).

### A two-state model of synaptic plasticity also exhibit heterosynaptic plasticity

**Figure 8:**
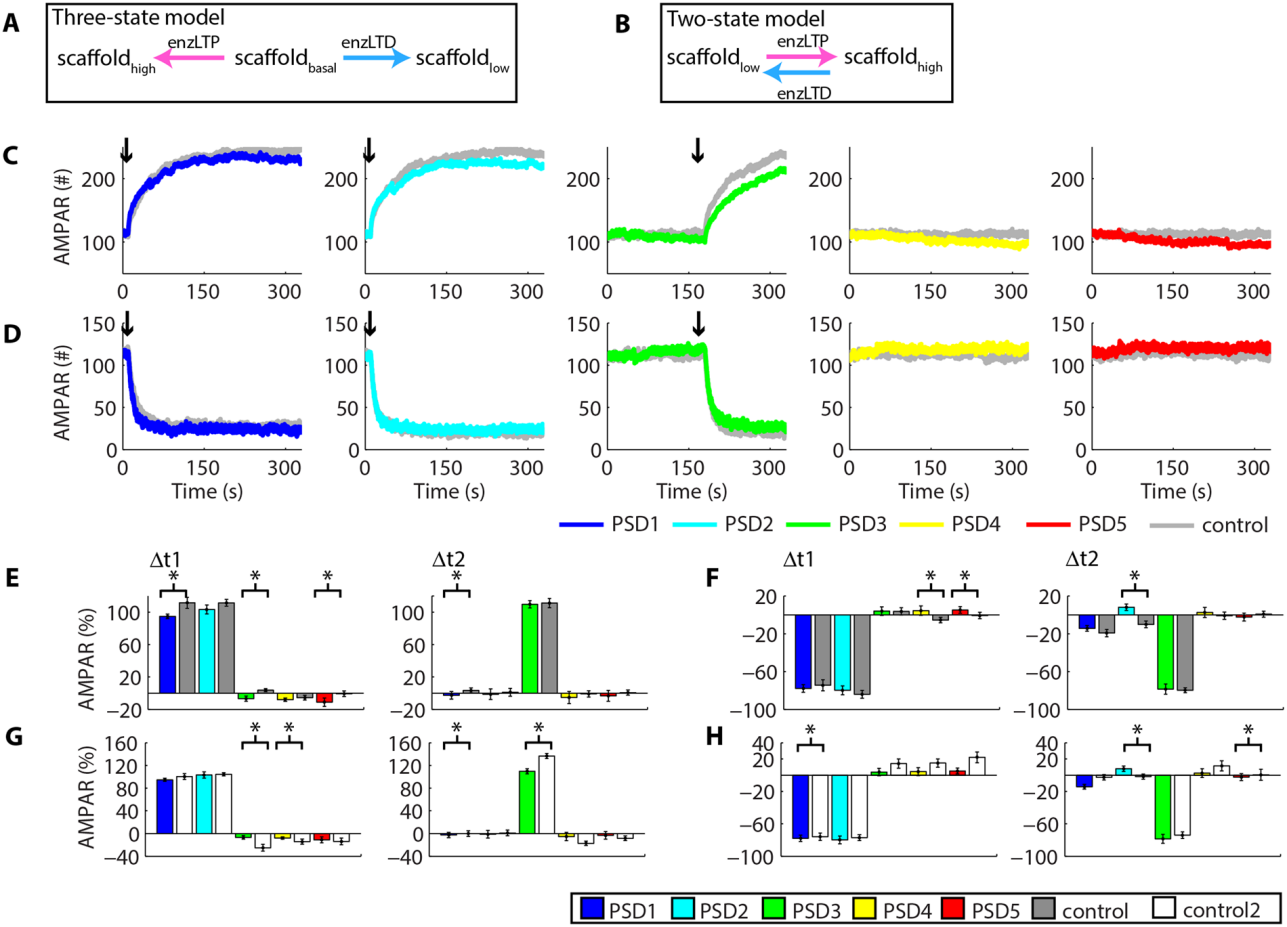
Heterosynaptic plasticity in a two-state model of synaptic plasticity. (**A,B**) Schematic representations of the three-state model **(A)** and the two-state model **(B)**. **(C)** Time courses of AMPARs at each synapse during prior LTP at PSD1 and PSD2, and posterior LTP at PSD3. All curves obtained for the control simulations are showed in Suppl. Fig. S9. **(D)** Time courses of AMPARs at each PSD during prior LTD at PSD1, PSD2, and posterior LTD at PSD3. **(E)** Alterations of AMPARs at each PSD caused by prior LTP (Δt1) and posterior LTP (Δt2) in comparison to control simulations. **(F)** Alterations of AMPARs at each PSD caused by prior LTD (Δt1) and posterior LTD (Δt2). **(G,H)** Comparisons between the results of the three-state model (control 2) and the two-state caused by prior and posterior LTP **(G)** and prior and posterior LTD **(H)**. Asterisks indicate statistically significant T-test comparisons (P < 0.05) with control simulations.

**Figure 9:**
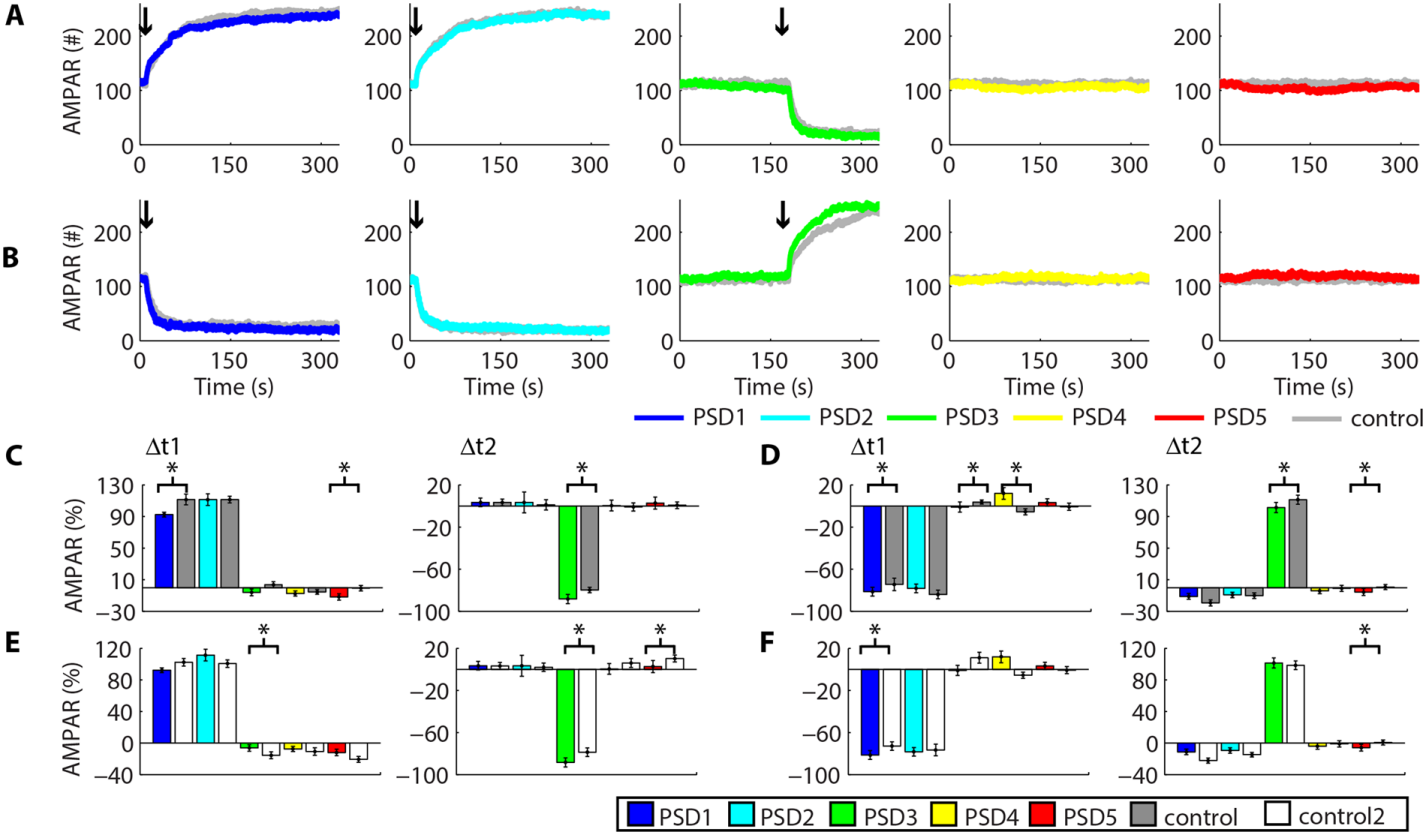
Heterosynaptic plasticity and the posterior induction of activity-driven synaptic plasticity in a two-state model. Time courses of AMPARs at each synapse for the prior inductions of LTP **(A)** or LTD (**B**) at PSD1 and PSD2 and the posterior induction of LTD **(A)** or LTP **(B)** at PSD3. **(C)** Alterations of AMPARs at each PSD caused by prior LTP (Δt1) and posterior LTD (Δt2) in comparison to control simulations. **(D)** Changes of AMPARs at each PSD caused by prior LTD (Δt1) and posterior LTP (Δt2) in comparison to control simulations. **(E,F)** Comparisons between the results of the three-state model (control 2) and the two-state model for prior LTP and posterior LTD **(E)** and prior LTD and posterior LTP **(F)**. Asterisks indicate statistically significant T-test comparisons (P < 0.05) with control simulations.

Next, we tested whether the heterosynaptic alterations observed could result from the assumptions used in our model. As mentioned previously, many models of synaptic plasticity are two-state systems (LTP and LTD) instead of the three-state system (LTP, LTD and basal state, Fig. 8A) that we simulated in this work and previously^28^. A two-state model must exhibit a basal state that represents LTP or LTD or a mixture of both processes. The last hypothesis requires high levels of balanced post-translational modifications involved with LTP/LTD at rest, but experimental evidence demonstrated low levels of phosphorylations involved with LTP during basal conditions^32^. Moreover, high levels of basal phosphorylation would require constant activation of kinases, but many data have indicated that kinase inhibitors do not alter the basal synaptic strength^33^. Nevertheless, we implemented a two-state model in which the phosphorylation of scaffold proteins by enzLTP changed their affinity to interact with AMPARs from low to high. The enzyme enzLTD dephosphorylated the scaffolds to restore their low affinity (Fig. 8B). The basal state consisted of a mixture of high (40% of the scaffolds) and low-affinity scaffolds (60% of the scaffolds). The high/low ratio was set to match approximately the number of synaptic AMPARs observed in the three-state model. Next, we simulated LTP at PSD1 and PSD2, and, posteriorly, at PSD3 (Fig. 8C). In a different set of simulations, we induced LTD at PSD1 and 2 and, posteriorly, at PSD3 (Fig. 8D). We measured the change in the number of AMPARs at each PSD between 10 s and 160 s (Δt1) caused by the first occurrences of synaptic plasticity. Next, we measured the variations of AMPARs between the induction of posterior LTP or LTD (180 s) and the end of the simulations (Δt2). These results were compared to control simulations without the induction of synaptic plasticity (PSD4 and PSD5), and with control simulations of LTP or LTD induced at a single synapse without prior plasticity (PSD1, PSD2, and PSD3). Our results demonstrated that the two-state model also exhibited heterosynaptic plasticity (Fig. 8D,E). Thus, LTP at PSD1 and PSD2 reduced the synaptic weight at PSD3 by 6.76% ± 3.1 (n = 5, control: 3.75% ± 1.9, n = 5, P < 0.05, T-test) and at PSD5 by 11.1% ± 5.2 (n = 5, control: −0.71% ± 3.4, n = 5, P < 0.05, T-test). LTD at PSD1 and PSD2 increased the population of AMPARs at PSD5 in 5.16% ± 3.5 (n = 5, control: −0.71% ± 3.4, n = 5, P < 0.05, T-test). However, prior LTP did not modulate posterior LTP (PSD3: 109.7% ± 4.5, n = 5, control: 111.2% ± 5.8, n = 5, P = 0.38). Similarly, prior LTD did not affect posterior LTD (PSD3: −78.5% ± 4.5, n = 4, control: −79.8% ± 2.6, n = 4, P = 0.14, T-test). Moreover, comparisons between the results of the two-state model and the three-state model (control2) showed little variations (Fig. 8G,H). However, prior LTD affected posterior LTP and vice-versa in our two-state model (Fig. 9A,B). As shown in Fig. 9C, prior LTP at PSD1 and PSD2 increased the magnitude of posterior LTD at PSD3 in the two-state model (PSD3: −88.3% ± 4.3, n = 5, control: −79.8% ± 2.6, n = 4, P < 0.05, T-test). Prior LTD reduced posterior LTP at PSD3 (PSD3: 101% ± 6.4, n = 5, control: 111.2% ± 5.8, n = 5, P < 0.05, T-test) in comparison to control simulations without previous LTD at nearby synapses (Fig. 9D). The comparisons with the three-state model confirmed that both models exhibited little variations, which consisted mainly of differences in the magnitude of heterosynaptic plasticity (Fig. 9E,F). Both models clearly indicate that prior synaptic plasticity can modulate posterior synaptic plasticity at vicinal synapses.

We also tested a two-state model with a basal state consisting of 50% high-affinity scaffolds and 50% low-affinity scaffolds, but the number of synaptic AMPARs at rest was higher than the number observed for the three-state model, so we did not perform additional statistical analysis for these results (Suppl. Fig. S10). However, the data obtained indicated the occurrence of heterosynaptic compensatory changes assessed through visual inspection (Suppl. Fig. S10). Additional simulations for two-state models using different sets of affinities also exhibited heterosynaptic plasticity (Suppl. Fig. S11).

## Discussion

In this work, we showed that changes in the number of synaptic AMPARs during LTP and LTD can promote heterosynaptic compensatory alterations that rescale the synaptic weights of non-stimulated synapses and modulate further inductions of activity-driven synaptic plasticity. In our system, LTP and LTD consisted of increases and decreases, respectively, in the synaptic populations of AMPARs due to changes in their affinities to interact with scaffold molecules^16,17^. Both the increase and the decrease of synaptic AMPARs relied heavily on the lateral diffusion of receptors. The system presented in this work is a simple implementation of LTP and LTD in comparison to other works^24,28,34,35^. Nevertheless, our results revealed several aspects of the dynamics of synaptic plasticity due to the incorporation of some basic features of biological systems that intrinsically promote stability. Biological processes are executed by finite entities. Hence, they are saturable and can exhibit intrinsic competition. The occurrences of LTP and LTD involving finite entities promoted compensatory heterosynaptic changes as an emergent property of our model. This is a sharp contrast with many phenomenological models of synaptic plasticity, which often require the implementations of multiple synaptic plasticity mechanisms with different time frames to achieve stable memory formation and recall^3,36^.

Inductions of synaptic plasticity in our system promoted compensatory changes of the weights of vicinal synapses. However, these changes were also balanced with the dendritic and intracellular pools of receptors. This setup allowed the synapses to rescale their weight through compensatory mechanisms without erasing the outcomes of the previous occurrences of activity-driven LTP and LTD, a problem observed in previous models of local synaptic plasticity^37^.

Results from a preceding computational model of local homeostatic synaptic plasticity predicted that the potentiation of a single spine could be achieved by the weakening of the adjacent spines^37^. In our model, we observed that the potentiation of a single dendritic spine and the weakening of the neighbouring spines are related to each other through the competition for available AMPARs. Nevertheless, the push-pull competition for AMPARs in our model could also play a role in the homeostasis of synaptic plasticity.

Many previous works have used computational models to investigate the mechanisms involved with AMPAR trafficking and have made several important predictions showing that the number of scaffold molecules and their affinities to interact with AMPARs play a central role in the accumulation of synaptic receptors, but they focused on the populations of AMPARs at single synapses^17,38–41^. To our knowledge, our model is the first to investigate how activity-driven synaptic plasticity at specific synapses affects the adjacent populations of synaptic AMPARs.

Due to the computational cost of our simulations, we were unable to determine the time window in which the heterosynaptic alterations are sustained in our system. However, we do not expect them to last more than few minutes. Though the initial change of AMPARs during LTP is mainly caused by lateral diffusion, the exocytosis of receptors replenishes the pools that act as the initial source of AMPARs^29,30^. Yet, factors such as spine density, the tortuosity of the dendritic segment, and other signalling processes might modulate the effects observed in our system. Consequently, our results can be combined with results of previous models and experimental works that have explored several different molecular processes involved in the structural stabilization of LTP and LTD and in further occurrences of synaptic plasticity^11–13,34,35^. The main mechanisms proposed for explaining local plasticity of multiple synapses are the synaptic tagging and capture and the crosstalk among multiple synapses caused by the intracellular diffusion of activated enzymes^11,35^. Based on our modelling results, we propose that heterosynaptic plasticity caused by lateral diffusion of AMPARs could work as an additional mechanism of local plasticity. Moreover, other dynamics aspects of the signalling networks involved with LTP and LTD and with the regulation of signalling systems can be used to expand the system presented here^28,35,42,43^.

Recent data have indicated that the potentiation of clusters of synapses might be a key event in memory formation^44,45^. Our results indicated that mechanisms of AMPAR trafficking involved with LTP and LTD could regulate the induction of activity-driven synaptic plasticity at nearby synapses and favour the formation of synaptic clusters. At least two mechanisms of our system contribute to these clusters formations. The occurrence of LTP promotes heterosynaptic depression at nearby synapses, which increases the number of scaffold molecules available to interact with AMPARs amplifying the percentage of change in the synaptic weight for posterior occurrences of LTP. Also, the heterosynaptic depression of non-stimulated synapses intensifies the synaptic weight of the potentiated synapses.

Synaptic clusters within the dendrites of biophysically inspired overlapping neurons with synaptic plasticity in neural networks can be used to store memory engrams^46^. Computation modelling studies of dendritic integration have proposed that the memory capacity of neurons is much larger if synaptic inputs are summed nonlinearly^47^. The heterosynaptic rules observed in our model can contribute for the nonlinear computation of the synaptic inputs to increase the memory capacity of models of neurons.

## Methods

We developed the reaction-diffusion computational model presented in this work using CellBlender and MCell^18–20^. The full description of the reactions and parameters used in the model is listed in Suppl. Table S1. The tridimensional structure simulated consisted of a small dendritic segment of a pyramidal CA1 hippocampal neuron with 2 µm of length and 0.5 µm of diameter^48^. We included manually 5 spines along the dendritic segment. Each dendritic spine consisted of a small cylindrical neck (approximately 0. 25 µm of length and 0.2 µm of diameter) connected to the spine head implemented using a modified sphere (0.5 µm of diameter^48^) with a flat top. The flat top of each spine head simulated its PSD.

At the beginning of the simulations, we released 1000 AMPARs randomly distributed on the dendritic segment membrane excluding the two lateral sections, which were implemented as totally reflective to the surface molecules. Each PSD had 300 copies of scaffold molecules. We set a perisynaptic region that surrounded each PSD and acted as a reflective barrier to the scaffold molecules to prevent their diffusion. This perisynaptic region also functioned as the EZ where the enzymes involved with the endocytosis and exocytosis of AMPARs were placed. In hippocampal neurons, the exact site for the endocytosis and exocytosis of AMPARs is a debatable issue^29,49^. For simplicity, we implemented both endocytosis and exocytosis of AMPARs mediated by a single protein, which we termed EEP. We included 10 copies of EEP per EZ^22^. We set the parameters for the endocytosis/exocytosis (k_f3_ and k_b3_) based on experimental data^50^. Other parameters (*k*_*f2*_ and *k*_*b2*_) were tuned to sustain a population of synaptic AMPARs around 100 copies per PSD. To simulate the pool of AMPAR_cyt_, we implemented a half-sphere mesh inside each spine head to model a single large endosome where we released 100 AMPARs at the start of the simulations (Fig. 2A,B). We set the top of each endosome as a permeable membrane. Thus, the receptors were free to diffuse out of the endosome and eventually react with the EEP molecules located on the perisynaptic membrane (Fig. 2C,D). The endosomes were the initial source of other intracellular molecules released inside specific spines during the simulations. The endosomes also prevented the diffusion of the cytoplasmic molecules of each dendritic spine. Thus, though the top membrane of the endosomes was permeable to the cytoplasmic molecules, the bottom membranes were impermeable and sealed the spine neck.

To simulate synaptic plasticity, we used the initial time to allow the system to reach steady-state before releasing enzLTP and enzLTD. These initial intervals (30 s for most simulations, and 50 s for the simulations of Figs 8 and 9, Suppl. Fig. S6, and Suppl. Figs S9 and S10) were suppressed from the analysis (Figs 3–9).

To simulate LTP and LTD, we implemented the phosphorylations of the scaffold molecules forming scaffold_LTP_ and scaffold_LTD_ catalyzed by enzLTP and enzLTD, two generic enzymes. The parameters for the phosphorylation reactions were chosen arbitrarily to allow fast phosphorylation of all the scaffold molecules at a given synapse (Supplementary Table S1). During the simulations of synaptic plasticity, scaffold_LTP_ reacted with AMPARs with an affinity 10-fold stronger than the control scaffold. The species scaffold_LTP_ corresponded to high-affinity scaffolds in Figs 8 and 9. The molecule scaffold_LTD_ (which corresponded to low-affinity scaffolds in Figs 8 and 9) interacted with AMPARs with a 10-fold weaker affinity. We implemented changes in the affinities using alterations in the rate constant for the dissociation of AMPAR bound to the phosphorylated scaffolds^28^.

In Figs 4–9, we computed the time courses of the populations of synaptic AMPARs from numerical simulations of 180 s (Figs 4 and 5) or 330 s (Figs 6–9) of the model dynamics. We ran 5 to 12 repetitions of each stochastic simulation using different random seeds. We calculated the mean ± standard deviation of the mean (STD) of the AMPARs at each PSD before and after the synaptic stimulation. We tested for significant differences from baseline (P < 0.05) between means using paired T-tests. For multiple comparisons between conditions, we performed one-way ANOVA tests using the software PAST 3. Tukey post hoc tests were used for accessing statistical significance for multiple comparisons.

## Electronic supplementary material

Supplementary Info
