# Supplementary material for: AMPA receptor trafficking and its role in heterosynaptic plasticity
Source: Sci Rep. 2018 Jul 9;8:10349. doi: 10.1038/s41598-018-28581-w (PMC6037747; doi:10.1038/s41598-018-28581-w)
Supplement: Supplementary file 1 — Supplementary Info [file 41598_2018_28581_MOESM1_ESM.pdf]

Supplementary material

Title: AMPA receptor trafficking and its role in heterosynaptic plasticity

Authors: Gabriela Antunes, Fabio M. Simoes-de-Souza

Filiation: Center for Mathematics, Computation and Cognition, Federal University of ABC, São Bernardo do Campo, SP, Brazil

Supplementary Fig. S1: Accumulation of synaptic AMPARs associated with scaffolds.
Supplementary Fig. S2: Accumulation of synaptic AMPARs associated with scaffolds is regulated by the rate constants used in the model.
Supplementary Fig. S3: Control simulations without synaptic plasticity.
Supplementary Fig. S4: Control simulations of LTP (arrows) only at PSD1 (A), PSD2 (B), PSD3 (C), and PSD4 (D).
Supplementary Fig. S5: Control simulations of LTD (arrows) only at PSD1 (A), PSD2 (B), PSD3 (C), and PSD4 (D).
Supplementary Fig. S6: The role of the number of scaffold molecules and free AMPARs on the results of the model.
Supplementary Fig. S7: Simulations of the model with a continuous flow of AMPARs through the lateral membranes of the dendritic segment.
Supplementary Fig. S8: Control simulations used in Fig. 6 and 7.
Supplementary Fig. S9: Control simulations used in Fig. 8-9.
Supplementary Fig. S10: A two-state model with basal state composed of 50% high-affinity scaffold and 50% low-affinity scaffolds also exhibited heterosynaptic plasticity for LTP and LTD induced at nearby synapses.
Supplementary Fig. S11: Heterosynaptic plasticity in two-state models of synaptic plasticity with different affinities.
Supplementary Table S1: Parameters of the model

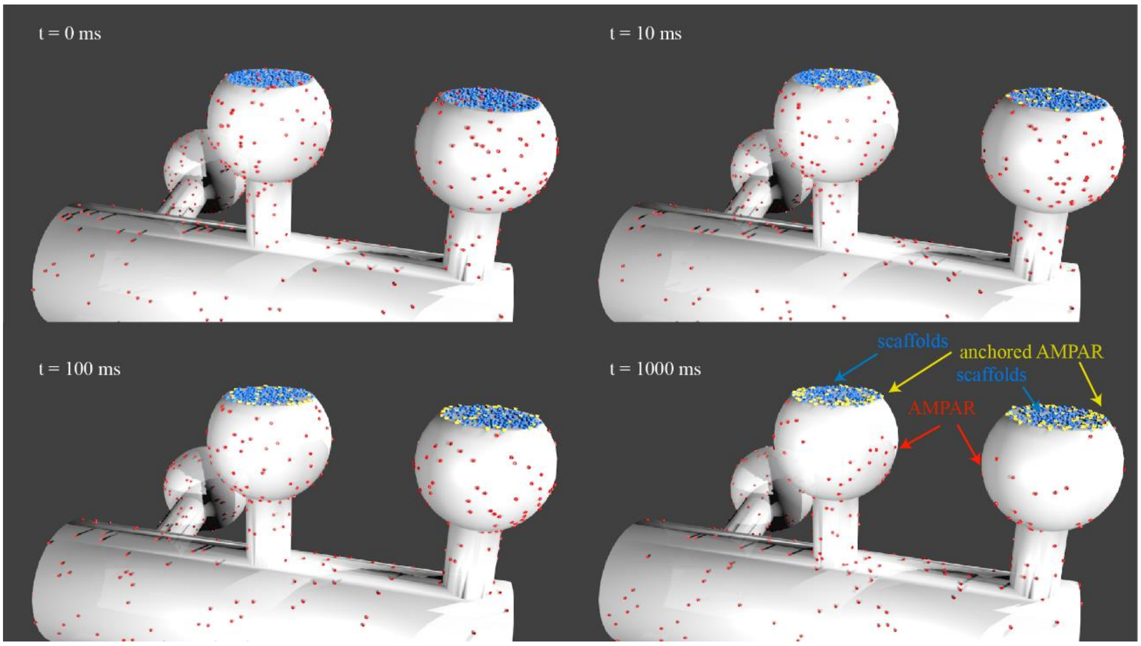

Supplementary Fig. S1: Accumulation of synaptic AMPARs associated with scaffolds. The anchored receptors are shown in yellow, free scaffold molecules are shown in blue and free AMPARs are shown in red.

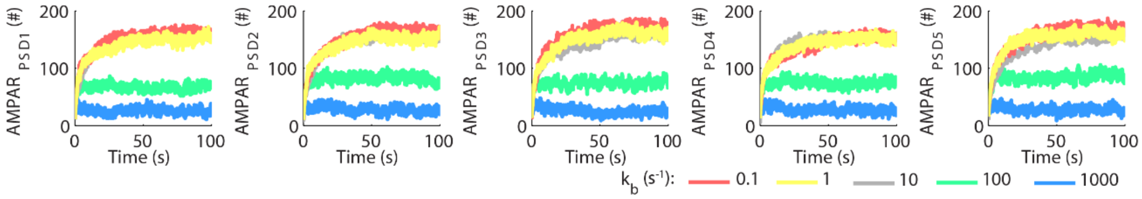

Supplementary Fig. S2: Accumulation of synaptic AMPARs associated with scaffolds is regulated by the rate constants used in the model. The rate constant for the dissociation of AMPARs from the scaffold molecules controlled the number of synaptic receptors stabilized at each PSD (number of scaffolds set as 200 per synapse).

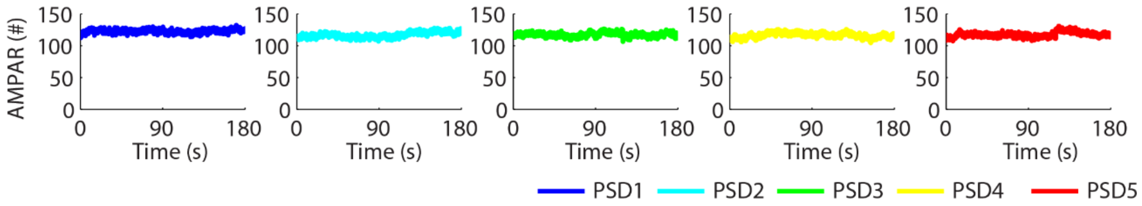

Supplementary Fig. S3: Control simulations without synaptic plasticity.

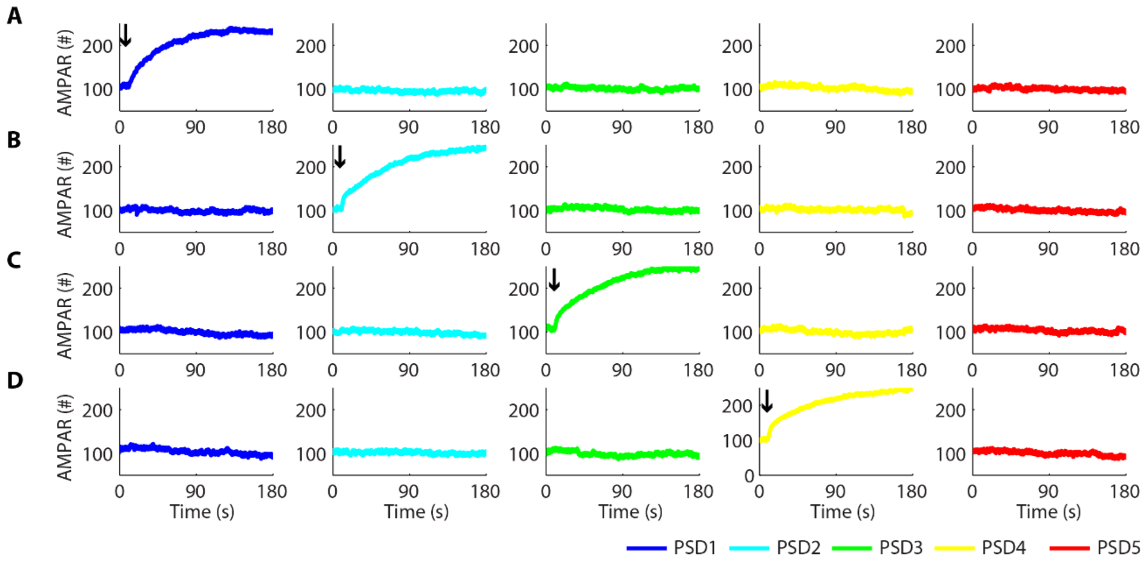

Supplementary Fig. S4: Control simulations of LTP (arrows) only at PSD1 **(A)**, PSD2 **(B)**, PSD3 **(C)**, and PSD4 **(D)**.

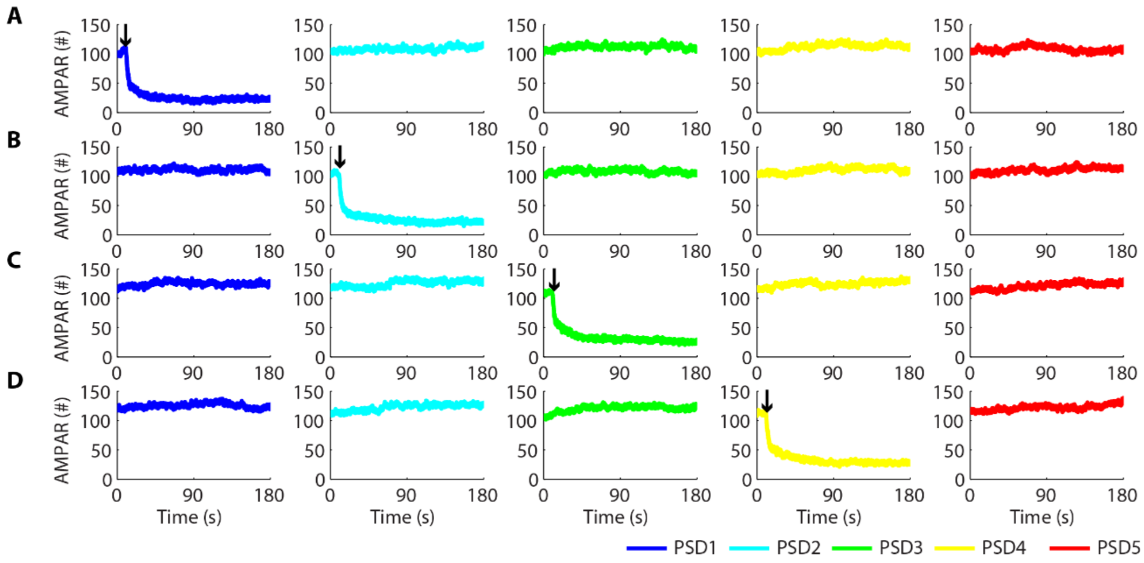

Supplementary Fig. S5: Control simulations of LTD (arrows) only at PSD1 **(A)**, PSD2 **(B)**, PSD3 **(C)**, and PSD4 **(D)**.

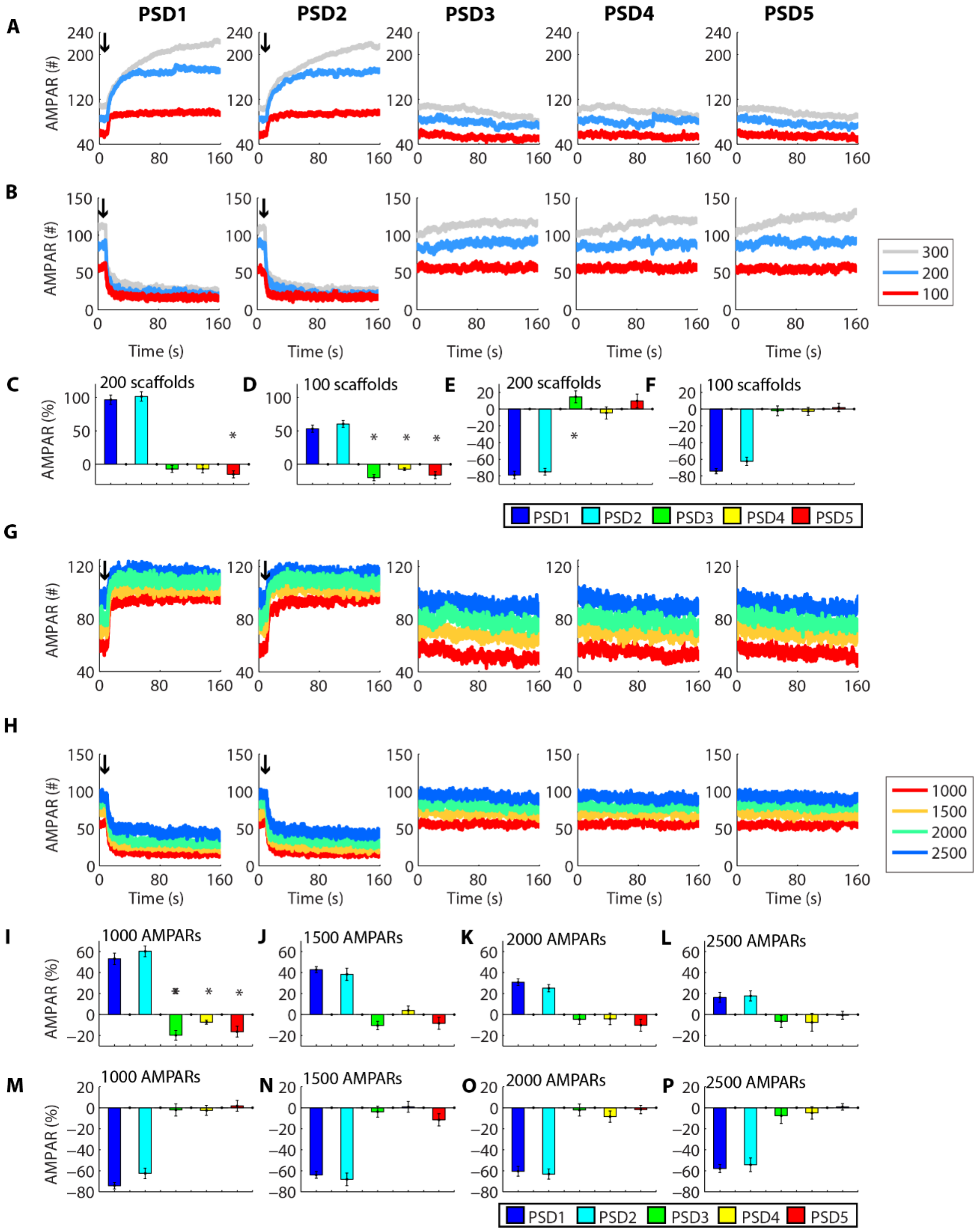

Supplementary Fig. S6: The role of the number of scaffold molecules and free AMPARs on the results of the model. Simulations of LTP **(A)** and LTD **(B)** at PSD1 and PSD2 for the model with 300 (control), 200 and 100 scaffolds per synapse. The arrows show the moment of synaptic plasticity induction. The changes in the number of scaffolds affected the basal number of synaptic AMPARs and the magnitude of the synaptic plasticity. **(C-F)** Variations of the number of AMPARs from the moment of

LTP **(C-D)** or LTD **(E-F)** induction to the end of the simulations. Simulations of LTP **(G)** and LTD **(H)** at PSD1 and PSD2 for the model with different number of free AMPARs released at the beginning of the simulations. For these simulations, each synapse had 100 scaffold molecules. **(I-P)** Variations of the number of AMPARs from the moment of synaptic plasticity induction to the end of the simulations for the results showed in G **(I-L)** and H **(M-P)**. The asterisks in **(C-F)** and **(I-P)** indicate the synapses that showed statistically significant heterosynaptic plasticity ($P < 0.05$, T-test).

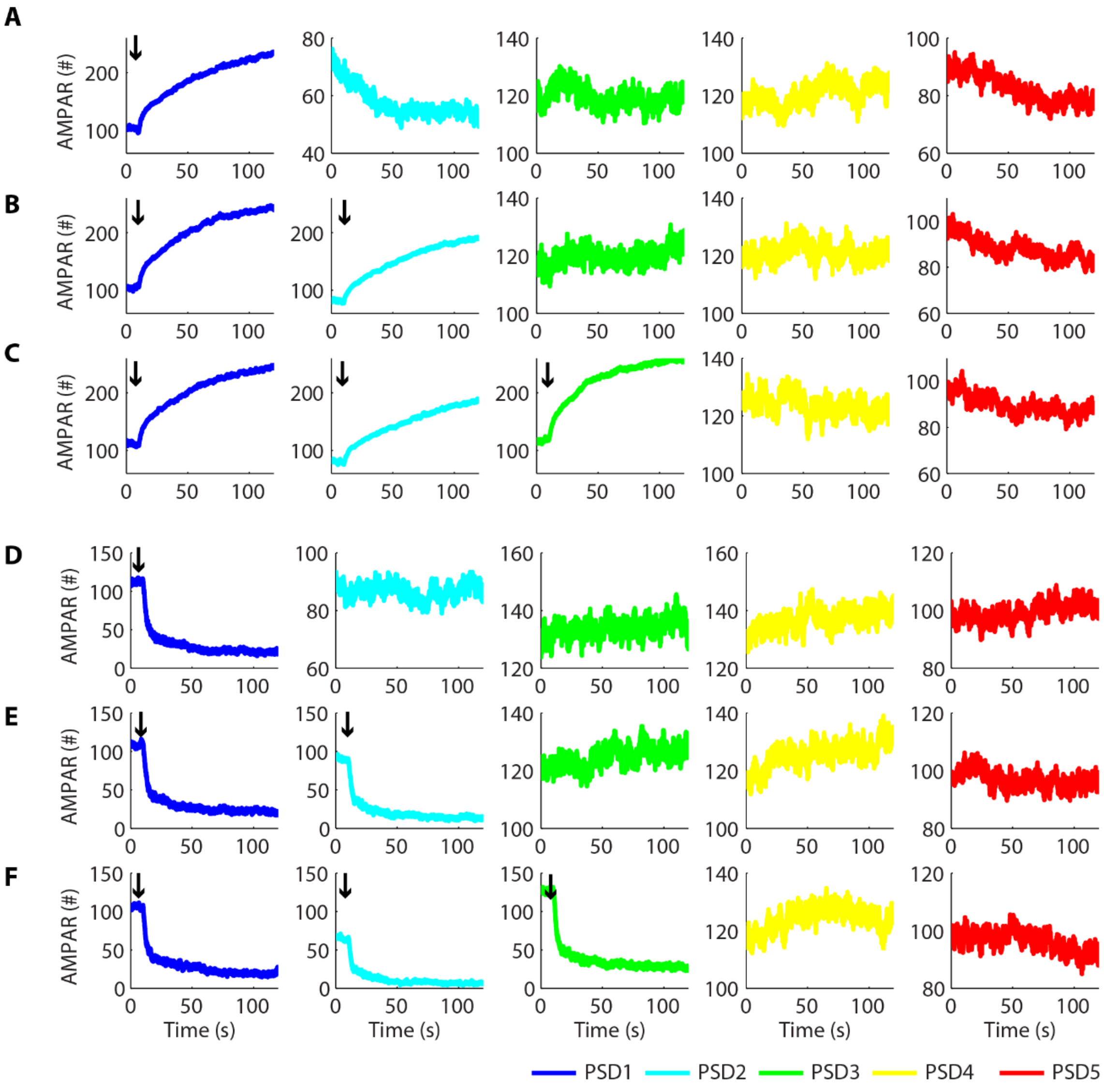

Supplementary Fig. S7: Simulations of the model with a continuous flow of AMPARs through the lateral membranes of the dendritic segment. To implement the flow of AMPARs through the membranes, we set both lateral membranes as absorptive and, at the edge of the lateral cross-sections, we simulated a constant production of AMPARs using a generic enzyme (enzyme → enzyme + AMPAR). A total of 20 enzymes were randomly distributed surrounding each lateral membrane. The rate constant of AMPAR production was set to 20 s^{-1}. The simulations showed great variability in the number of AMPARs at each synapse, but the model still exhibited heterosynaptic depression during LTP **(A-C)** and heterosynaptic potentiation during LTD **(D-E)** at nearby synapses. The arrows indicate LTP and LTD inductions at specific synapses.

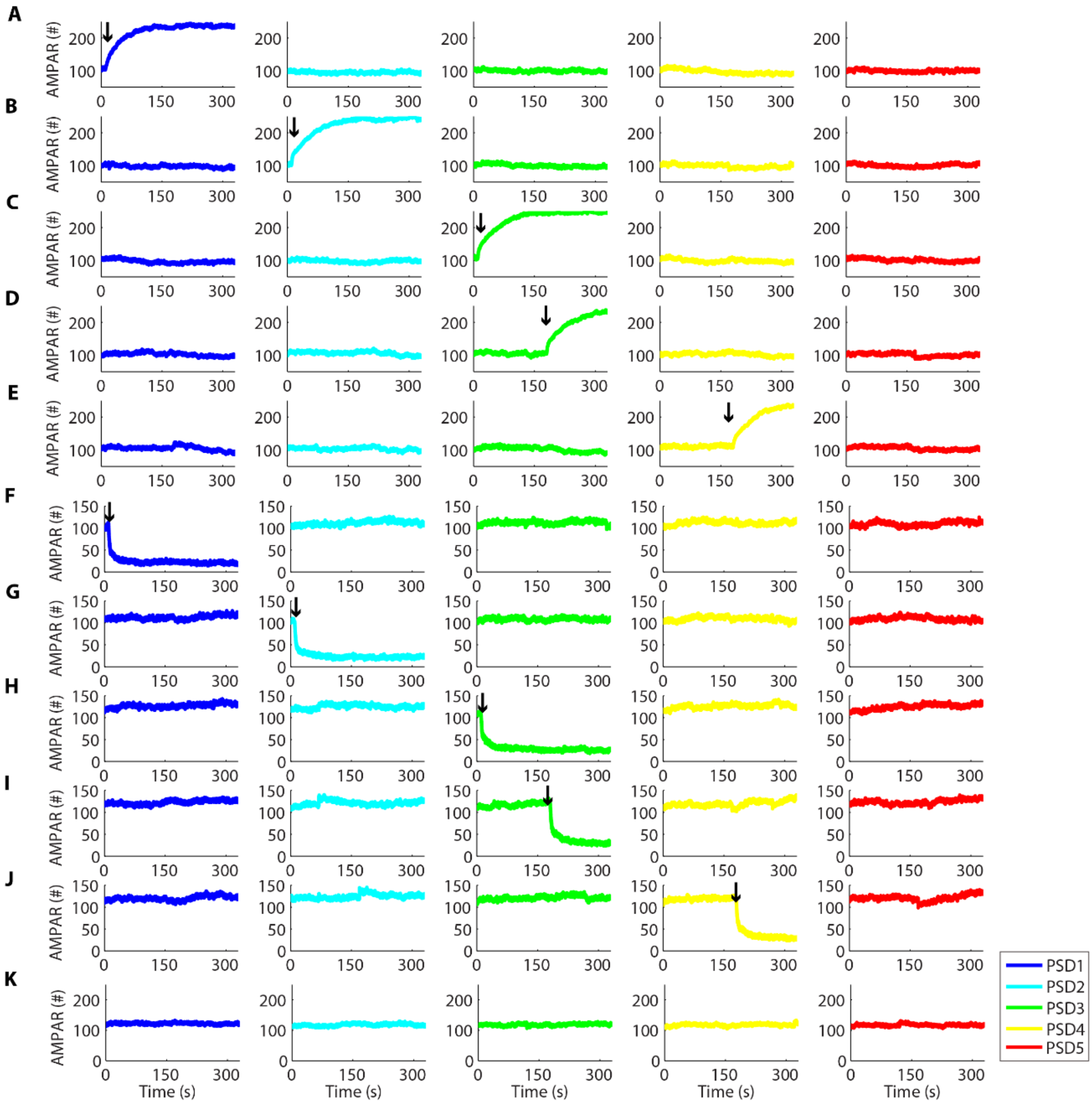

Supplementary Fig. S8: Control simulations used in Fig. 6 and 7. **(A-E)** Control simulations of the induction of LTP (arrows) only at PSD1 **(A)**, PSD2 **(B)**, PSD3 **(C and D)**, and PSD4 **(E)**. **(F-J)** Control simulations of LTD (arrows) at PSD1 **(F)**, PSD2 **(G)**, PSD3 **(H and I)**, and PSD4 **(J)**. **(K)** Control simulations without synaptic plasticity. Some curves were also used as a control for Fig. 4 and 5 and were plotted previously in Supplementary Fig. S3-S5.

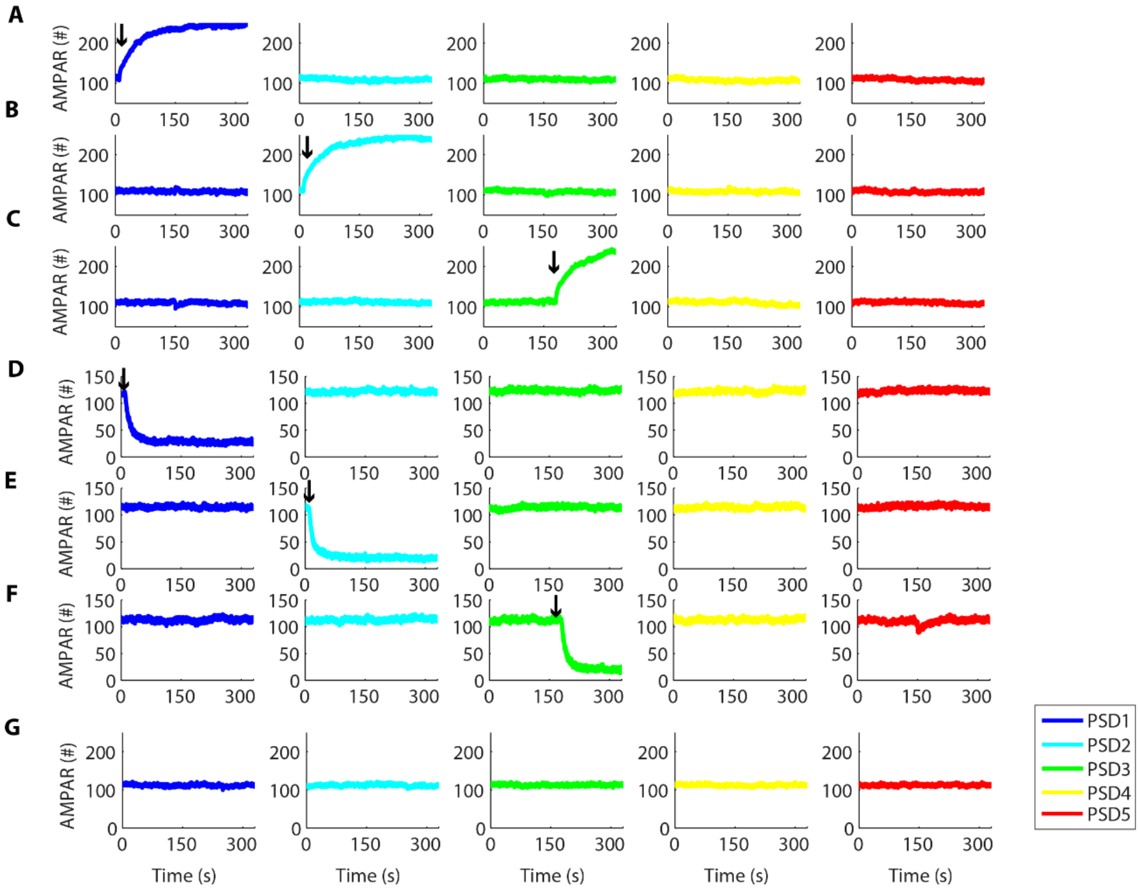

Supplementary Fig. S9: Control simulations used in Fig. 8-9. **(A-C)** Control simulations of the induction of LTP (arrows) only at PSD1 **(A)**, PSD2 **(B)** and PSD3 **(C)**. **(D-F)** Control simulations of LTD (arrows) at PSD1 **(D)**, PSD2 **(E)**, and PSD3 **(F)**. **(G)** Control simulations without synaptic plasticity.

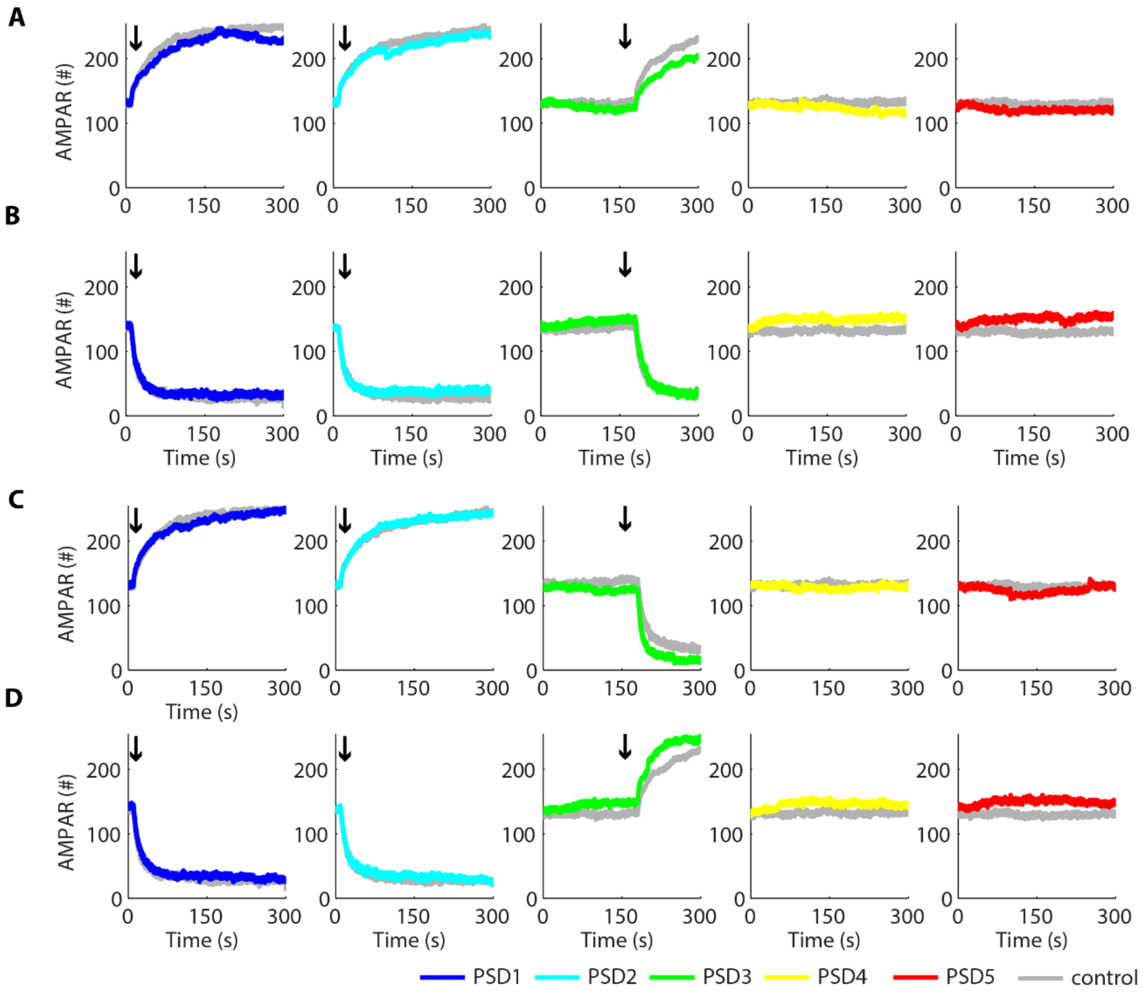

Supplementary Fig. S10: A two-state model with basal state composed of 50% high-affinity scaffolds and 50% low-affinity scaffolds also exhibited heterosynaptic plasticity for LTP and LTD induced at nearby synapses. **(A)** Prior LTP at PSD1 and PSD2 and posterior LTP at PSD3. **(B)** Prior LTD at PSD1 and PSD2 and posterior LTD at PSD3. **(C)** Prior LTP at PSD1 and PSD2 and posterior LTD at PSD3. **(D)** Prior LTD at PSD1 and PSD2 and posterior LTP at PSD3. The arrows show LTP/LTD induction.

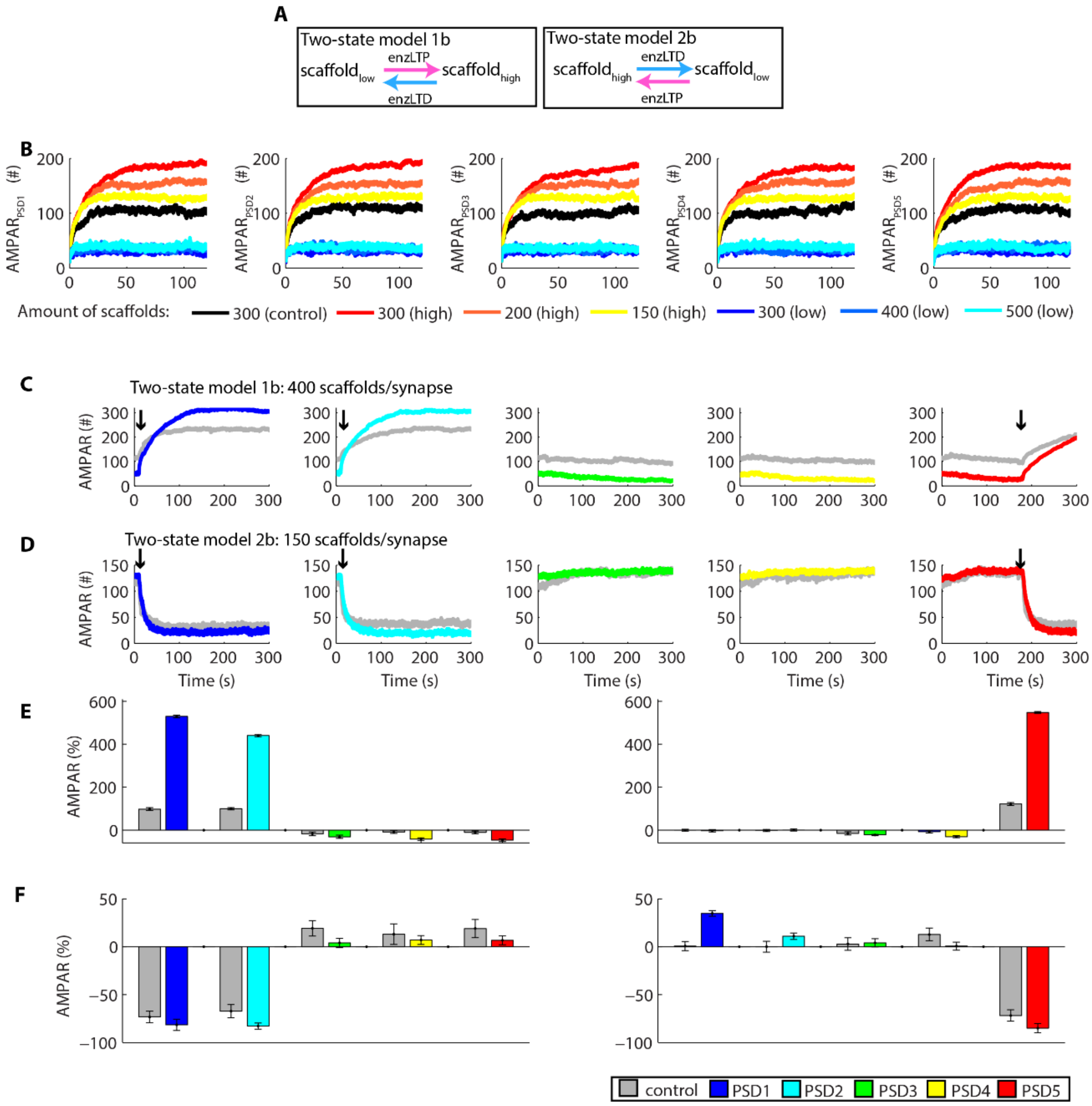

Supplementary Fig. S11: Heterosynaptic plasticity in two-state models of synaptic plasticity with different affinities. **(A)** To verify whether our results with a two-state model were caused by the affinities used, we performed simulations starting from a basal state composed solely by low affinity scaffolds (two-state model 1b) or only high affinity scaffolds (two-state model 2b). **(B)** As the change in the affinities for the interaction of AMPARs with scaffolds alters the number of synaptic AMPARs, initially we performed simulations to verify the amounts of scaffolds that could promote approximately the same amount of AMPARs per synapse (around 100-120 receptors) used in our control model. In this panel, high indicates high affinity, low is the low affinity and control is the basal affinity used in the other models. Our results showed that changing the affinity from moderate to low drastically reduced the amount of synaptic AMPARs even when we increased the number of scaffolds, which indicated

that, for these simulations, the number of AMPARs available for binding was the limiting factor. We arbitrarily chose 400 anchors as the initial condition of our simulations for the two-state model 1b. For the two-state model 2b, we set 150 scaffold molecules as the amount of free scaffold released at the beginning of the simulations. **(C)** We used the two-state model 1b to simulate LTP of two synapses simultaneously and a posterior induction of LTP at a nearby spine. Results obtained with the control three-state model are shown in grey. The results for the two-state model 1b showed intense heterosynaptic alterations caused by all the occurrences of LTP tested. **(D)** The two-state model 2b was used to simulate the induction of LTD simultaneously at two synapses and, posteriorly, at another adjacent synapse. The time courses observed were very similar to the control three-state model, but the magnitudes of the homosynaptic and heterosynaptic alterations were different. **(E-F)** Quantifications of the changes of synaptic AMPARs caused by the first ($\Delta t1 = 120$ s, from 10 s to 130 s) and the posterior ($\Delta t2 = 120$ s, from 180 s to 300 s) inductions of synaptic plasticity.

Supplementary Table S1: Parameters of the model.

**Species/reactions**	**Parameter**
Total number of AMPARs on the mesh membrane	1000
Number of cytosolic AMPARs per spine	100
Number of scaffold molecules per spine	300
Number of endocytic/exocytic protein (EEP) per spine	10
Scaffold diffusion coefficient	0.001 $\mu m^2.s^{-1}$
Diffusion coefficient of AMPAR bound to scaffold	0.001 $\mu m^2.s^{-1}$
Diffusion coefficient of free AMPAR	0.05 $\mu m^2.s^{-1}$
$scaffold + AMPAR \underset{k_{b1}}{\overset{k_{f1}}{\rightleftharpoons}} scaffold.AMPAR$	k_{f1} = 1 $\mu m^2.molecule^{-1}.s^{-1}$<br>$k_{b1}$ = 100 s^{-1}
$EEP + AMPAR_{EZ} \underset{k_{b2}}{\overset{k_{f2}}{\rightleftharpoons}} EEP\_AMPAR_{EZ}$	k_{f2} = 0.005 $\mu m^2.molecule^{-1}.s^{-1}$<br>$k_{b2}$ = 1 s^{-1}<br>$AMPAR_{EZ}$ refers to AMPARs located at EZ, EEP_AMPAR_{EZ} is the complex EEP bound to $AMPAR_{EZ}$, and $AMPAR_{cyt}$ refers to the cytosolic AMPARs. The parameters k_{f2} and k_{b2} were tuned to sustain a population of synaptic AMPARs around 100 copies per PSD.
$EEP + AMPAR_{EZ} \underset{k_{b3}}{\overset{k_{f3}}{\rightleftharpoons}} EEP + AMPAR_{cyt}$	k_{f3} = 0.1667 s^{-1}<br>k_{b3} = 0.01667 $\mu mol^{-1}.L.s^{-1}$<br>We set k_{f3} and k_{b3} based on experimental data (see main text).
$enzLTP + scaffold \underset{k_{b4}}{\overset{k_{f4}}{\rightleftharpoons}} enzLTP.scaffold$	k_{f4} = 1 $\mu mol^{-1}.L.s^{-1}$<br>k_{b4} = 2 s^{-1}<br>The term *enzLTP.scaffold* refers to the complex formed

	by the scaffold associated to enzLTP
$enzLTP.scaffold \xrightarrow{k_{cat4}} enzLTP + scaffold_{LTP}$	k_{cat4} = 8 s^{-1}<br>The term $scaffold_{LTP}$ indicates the scaffold phosphorylated by enzLTP
$enzLTD + scaffold \underset{k_{b5}}{\overset{k_{f5}}{\rightleftharpoons}} enzLTD.scaffold$	k_{f5} = 1 $\mu mol^{-1}.L.s^{-1}$<br>k_{b5} = 2 s^{-1}<br>The term *enzLTD.scaffold* refers to the complex formed by the scaffold associated to enzLTD
$enzLTD.scaffold \xrightarrow{k_{cat5}} enzLTD + scaffold_{LTD}$	k_{cat5} = 8 s^{-1}<br>The term $scaffold_{LTD}$ indicates the scaffold phosphorylated by enzLTD
$scaffold_{LTP} + AMPAR \underset{k_{b1}/10}{\overset{k_{f1}}{\rightleftharpoons}} scaffold_{LTP}.AMPAR$	k_{f1} = 1 $\mu m^2.molecule^{-1}.s^{-1}$<br>$k_{b1}$ = 100 s^{-1}
$scaffold_{LTD} + AMPAR \underset{10k_{b1}}{\overset{k_{f1}}{\rightleftharpoons}} scaffold_{LTD}.AMPAR$	k_{f1} = 1 $\mu m^2.molecule^{-1}.s^{-1}$<br>$k_{b1}$ = 100 s^{-1}
